# Blood host preferences and competitive inter-species dynamics within an African malaria vector species complex inferred from signs of animal activity around aquatic larval habitats

**DOI:** 10.1371/journal.pone.0344670

**Published:** 2026-03-27

**Authors:** Katrina A. Walsh, Deogratius R. Kavishe, Lily M. Duggan, Lucia J. Tarimo, Rogath V. Msoffe, Manase Elisa, Nicodem J. Govella, Markus P. Eichhorn, Emmanuel W. Kaindoa, Fidelma Butler, Gerry F. Killeen

**Affiliations:** 1 School of Biological Earth & Environmental Sciences, University College Cork, Cork, Republic of Ireland; 2 Sustainability Institute, University College Cork, Cork, Republic of Ireland; 3 Department of Environmental Health and Ecological Sciences, Ifakara Health Institute, Off Mlabani Passage, Ifakara, Morogoro, Tanzania; 4 College of Forestry, Wildlife and Tourism, Sokoine University of Agriculture, Morogoro, Tanzania; 5 Nyerere National Park, Tanzania; Clinton Health Access Initiative, UNITED STATES OF AMERICA

## Abstract

**Background:**

The feeding behaviours of the malaria vector *Anopheles arabiensis*, and its competitive relationships with other sibling species within the *Anopheles gambiae* complex, remain largely unexplored within well conserved natural ecosystems, where its known preferred hosts are scarce or absent.

**Methods:**

Potential aquatic habitats were surveyed for *An. gambiae* complex larvae across a gradient of natural ecosystem integrity in southern Tanzania, encompassing fully domesticated human settlements, a partially encroached Wildlife Management Area (WMA), and well conserved natural ecosystems within Nyerere National Park (NNP). Direct observations, tracks, spoor and other signs of human, livestock or wild animal activity around these water bodies were recorded as indirect indicators of potential blood source availability.

**Findings:**

While only *An. arabiensis* was found in fully domesticated ecosystems, its non-vector sibling species *An. quadriannulatus* occurred in conserved areas and dominated the most intact natural ecosystems. Proportions of larvae identified as *An. arabiensis* were positively associated with human and/or cattle activity and negatively associated with distance inside NNP and away from human settlements. Proportions of *An. quadriannulatus* were positively associated with activities of impala, warthog and possibly bushpig, implicating them as likely preferred blood hosts. While abundant impala and lack of humans or cattle in intact acacia savannah within NNP apparently allowed it to dominate *An. arabiensis*, presence of warthog seemed to provide it with a foothold in miombo woodlands of the WMA, despite encroachment there by people and livestock. While this antelope and suid are essentially unrelated, both are non-migratory residents of small home ranges with perennial surface water, representing potential hosts for *An. quadriannulatus* that are widespread across extensive natural ecosystems all year round. Despite dominance of *An. quadriannulatus* in well-conserved areas, *An. arabiensis* was even found in absolutely intact natural environments > 40km inside NNP, suggesting it can survive on blood from one or more unidentified wild species. Such self-sustaining refuge populations of *An. arabiensis* inside conservation areas, supported by wild blood hosts that are fundamentally beyond the reach of insecticidal interventions targeted at humans or livestock, may confound efforts to eliminate this key malaria vector. However, they might also enable insecticide resistance management strategies that could restore the effectiveness of pyrethroids in particular. This new approach to indirectly identifying commonly utilized blood sources may also be applicable to an unprecedented diversity of zoophagic mosquitoes, enabling incrimination of possible bridge vector species capable of mediating pathogen spillover from wildlife reservoirs into livestock and/or human populations.

## Introduction

The *Anopheles gambiae* complex consists of at least eight distinct sibling mosquito species that occupy overlapping ecological niches spanning most of sub-Saharan Africa [1–3]. Several of these sibling species act as highly efficient vectors of human malaria because they often feed upon people [4–7]. Following scale up of long-lasting insecticidal nets (LLINs) and indoor residual spraying (IRS) to protect vulnerable human populations against malaria transmission, the nominate species within this complex, *An. gambiae* Giles*,* was eliminated from large tracts of Africa over a remarkably brief period of a few short years, while its more zoophagic sibling species *An. arabiensis* proved much more resilient and persistent [8,9,10]. The subsequent dominance of the complex by *An. arabiensis* in many parts of Africa arises from a suite of phenotypically plastic behavioural traits that allow this far less exclusively human-dependent vector to minimize its exposure to the active ingredients of LLINs and IRS [11], thereby mediating persistent residual malaria transmission [12–15].

While *An. gambiae s.s.* feeds almost exclusively on human blood, *An. arabiensis* also feeds readily upon cattle [4,16,17]. Furthermore, as they do not appear to acquire bloodmeals from any other animal in domesticated landscapes, *An. arabiensis* appears strongly specialized to feed flexibly upon either or both of these host species, their exact choices depending closely upon their relative availabilities at scales as fine as a few metres [18,19]. Not only do zoophagic habits render mosquitoes less dependent upon human blood [7,12,13,20], such evolutionary adaptation to feeding upon animals is often associated with outdoor-biting, outdoor resting, early-exiting and crepuscular feeding behaviours [7,13,21]. *An. arabiensis* exhibits all of these behavioural traits, allowing it to evade exposure to insecticidal LLIN and IRS even when it does encounter humans [7,11,13,22]. Synergy between all these evasive behaviours make *An. arabiensis* notoriously resilient against attack with LLINs and IRS [11,22], and consequently an important vector of persistent residual malaria transmission [7,12,13].

*An. arabiensis* may also be able to escape pesticide pressure entirely by living in places where humans, livestock and agriculture are essentially absent, although the evidence to date remains ambiguous. While the known host preferences [4,16,17,19] and associated evasive behaviours exhibited by *An. arabiensis* [7,11] are consistent with adaptation to domesticated land use patterns [23], *An. arabiensis* populations have also been identified inside African conservation areas. For example, adult *An. arabiensis* mosquitoes were caught during a study inside Mana Pools National Park, Zimbabwe [24], although these experiments were completed at a permanent research camp with a small resident population of cattle. Similarly, while an *An. arabiensis* population has been documented at the Malahlapanga springs inside Kruger National Park in South Africa [25–27], and it has been suggested that this malaria vector may be surviving on blood meals from wild animals rather than their known preferred human and cattle hosts [25], this survey site was < 20 km from the park boundary and adjacent villages. It therefore remains unclear whether *An. arabiensis* can exploit alternative hosts in wild areas.

While on the one hand, the portfolio effect arising from such wild populations living far away from vector control interventions and other anthropogenic pesticide pressures could bolster this vector population against conventional efforts to control and eliminate it [28], it could also enhance the effectiveness of long term insecticide resistance management strategies by providing refugia against selective pressure [29–31]. If indeed such wild refugia populations of *An. arabiensis* do exist inside conserved wilderness areas, they might consequently retain original wild type traits for susceptibility to public health insecticides, particularly pyrethroids. Physiological resistance to the pyrethroids, arising from near-exclusive reliance upon this insecticide class for LLINs and IRS, has clearly undermined progress to date and now threatens a catastrophic resurgence of malaria-related morbidity and mortality [32–40]. The persistence of such genetically diverse populations of *An. arabiensis* inside conserved wilderness areas could therefore represent an invaluable form of natural capital [41–43], which could potentially be exploited by astute [29–31], long-overdue pre-emptive insecticide resistance management strategies [40,44]. Indeed, even reactive strategies to reverse selection trends towards resistance may be feasible [45–49] and could be enhanced by such natural reservoirs of standing genetic variation [29–31].

The *a priori* objective of this study was therefore to determine whether this key vector of residual malaria transmission [7,11–13,15] does indeed survive in such wild refugia by feeding upon one or more species of wild mammal. Fortuitously, this study also revealed the presence of *An. quadriannulatus,* another zoophagic, arid-adapted member of the *An. gambiae* complex of negligible relevance to malaria transmission [2–4,16,50–52] co-existing with *An. arabiensis* inside conserved natural ecosystems. This study therefore evolved over the course of its implementation, to also investigate the major drivers that allow these sibling species to co-exist in a finely-balanced competitive relationship.

## Methods

### Study area and design

This study was conducted in the Kilombero valley in the Morogoro region of southern Tanzania (S1 Appendix), where the local malaria transmission systems and vector populations have been exceptionally well-characterised [53–65].

**Fig 1:**
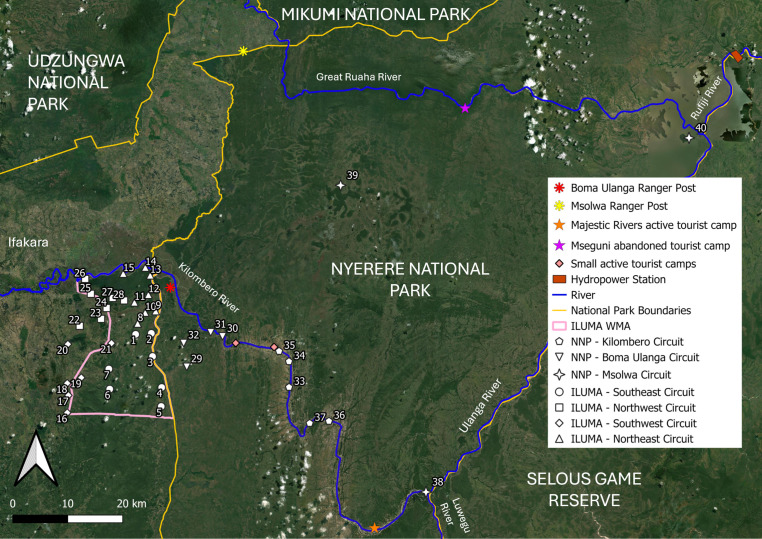
Map of the environmentally heterogeneous study area, ranging from fully domesticated human settlements just outside the western border of the Ifakara-Lupiro-Mangula Wildlife Management Area (ILUMA WMA [66,67]) through to well conserved natural ecosystems within Nyerere National Park (NNP) to the east of it, displaying the distribution of suitable camping locations used as the sampling frame for all the surveys described in this report. Each of the 40 *camps* detailed in S1 Table.1 in S1 Appendix are illustrated in the geographic context of the boundaries of the ILUMA WMA and NNP, with camp number 1 being the central main camp Msakamba [69]. Copyright © Esri. All rights reserved. Map layers were produced using base maps obtained from OpenStreetMap® under the Open Database License.

At the outset, the study design was centred around the Ifakara-Lupiro-Mangula Wildlife Management Area (ILUMA WMA) but was then later extended deep into Nyerere National Park (NNP), spanning diverse natural landcover and habitat types [66–68]. The selected study area (Fig 1) represents a geographical gradient of natural ecosystem integrity ranging from fully domesticated land uses in the west to completely intact, well conserved natural ecosystems to the east [66,67]. Note, however, that this study reports on surveys of human, livestock and wildlife activities that went far deeper into NNP than previous reports [66,67].

This environmentally heterogeneous study area was considered fundamental to the study objective as it captures a wide range of mammalian species abundance and diversity available to host-seeking adult mosquitoes as potential sources of blood. At one end of the gradient, humans and cattle are abundant around permanent settlements and domesticated land. At the other end of the gradient, it has been suggested that wild animals, particularly bovids, may act as suitable alternative hosts for wild *An. arabiensis* populations in wilderness areas [24–26,60], such as the well conserved areas of ILUMA WMA and NNP. Furthermore, at the time of the study, ILUMA WMA encompassed varying degrees of human disturbance [67,70] and so the availabilities of cattle, human and wildlife species as potential blood sources was expected to vary considerably across fine geographical scales.

However, over the course of this study, it was noted that several small but permanently occupied ranger posts and tourist camps inside NNP were scattered along the banks of the Kilombero and Ulanga Rivers, including the Majestic Rivers fishing camp only 11 km upstream from camp 38 (Fig 1). While these minor outposts were all very small (Between 2 and 30 occupants at any given time), they nevertheless represented perennial sources of human blood that are credibly within the regular flight ranges of *Anopheles* mosquitoes [71,72]. Therefore, at the very end of this study, one far less structured but more purposive impromptu survey was conducted at *Mseguni* (Fig 1), a long-abandoned hunting camp where the escorting park rangers indicated absolutely no humans had been present over the previous two years other than their occasional patrols.

This study was carried out using a rolling cross-sectional design [66,67] with four rounds of surveys encompassing a total of 40 defined locations, referred to herein as *camps,* over the course of two years (Fig 1 and Table S1.1 in S1 Appendix). Camps were carefully selected to include a range of ecosystem integrity states across three distinct landcover types; evergreen groundwater forest, moist deciduous miombo woodland savanna, and dry acacia savanna. However, the exact position of each camp was ultimately determined by the year-round availability of perennial surface water for larval occupancy surveys, and also for cooking, drinking and washing by the survey team in the absence of regular vehicle support.

Originally, a total of 28 camp locations were identified in or immediately to the west of the ILUMA WMA, most of which were sampled over each of 3 distinct survey rounds completed in 2022. However, no camp within ILUMA WMA lacked any signs of human disturbance and only a few remained relatively intact and undisturbed (Table S1.1 in S1 Appendix). Considering this and the unexpected identification of *An. quadriannulatus*, four new camps inside NNP were added at the end of the third round and a further eight for the fourth and final round of surveys between February and July 2023, spanning the whole wet season and the beginning of the dry season for that calendar year.

Camps were given a unique number and grouped into circuits based on their geographic proximity to one another (Fig 1). Most circuits were completed on foot, starting and ending at a central permanent camp (Camp 1 in Fig 1) that was established as the hub for all scientific and logistical processes in the field [69].

To determine if wild populations of *An. arabiensis* exist and survive by feeding on specific wild animals, aquatic larval habitat occupancy was investigated at each camp and associated with mammalian activity surrounding those larval habitats as described below.

### Surveys of mosquito larvae in perennial surface water bodies

Quantitative larval surveys were carried out within a 2 km radius of each camp, as explained in detail in S2 Appendix and summarized as follows. The upper distance limit of 2 km from the camp location was to prevent geographic overlap between neighbouring camp locations and minimise spatial autocorrelation effects. Surveys were initiated at the closest water body and carried out for a maximum of 4 hours to mitigate against investigator fatigue and maintain optimal data collection. An experimental design form template (EDSO1) adapted from a standardised mosquito collection informatics platform [73] was used characterise aquatic habitats and record occupancy by any *Anopheles* and by members of the *An. gambiae* complex specifically (Table S2.1 in S2 Appendix).

A sample of water, informally referred to in the field as a *dip*, was taken by briefly submerging a standard white 350 ml dipper just below the water surface into the habitat at a 45-degree angle. Dips were taken along the waterbody perimeter and were methodically numbered and spaced according to estimates of the habitat perimeter and other criteria outlined in S2 Appendix. Dips were inspected for the presence or absence of early and/or late instar *An. gambiae* complex larvae, distinguished from other anophelines based on a characteristic white collar immediately behind the head [74] (S2 Fig.3 in S2 Appendix; S3 Appendix), and recorded in the EDSO1 form.

During survey rounds 1–3, larvae were collected and returned to the central camp alive [69] to be reared to adults for a corresponding study on insecticide resistance before being identified to species level. However, it was decided that Polymerase-Chain-Reaction (PCR) tests on the reared adult mosquitoes were unlikely to give robust representations of sibling species composition of the sampled larvae because of expected survival biases in the rearing process [75,76].

Therefore, during the fourth survey round, the field-identified *An. gambiae* complex larvae from a singular aquatic habitat were immediately preserved as a *batch sample* [73] into a 50 ml tube filled with ethanol using a clean, disposable plastic pipette. A target of ten batch samples per camp was set *a priori*, to enable robust quantification and statistical evaluation of population composition heterogeneity around each camp location. Each habitat-specific batch sample was labelled with the camp number, the serial number and form-row number on the EDSO1 form and given a *breeding site identification number* from 1 to 10, so that each batch sample could be traced to a particular aquatic habitat from a particular camp. The individual specimens within each sample were then identified to sibling species level by PCR analysis [77].

In the case of the impromptu, far less structured survey at *Mseguni* (Fig 1) at the very end of the study, the team simply looked purposively for as many *An. gambiae* complex larvae as they could find in any habitats within a few kilometres of that camp over one working day. A single batch of 148 larvae suspected to be *An. gambiae* complex were collected and returned to the laboratory for species identification by PCR.

### Surveys of wildlife, livestock and human activities around perennial surface water bodies

**Fig 2:**
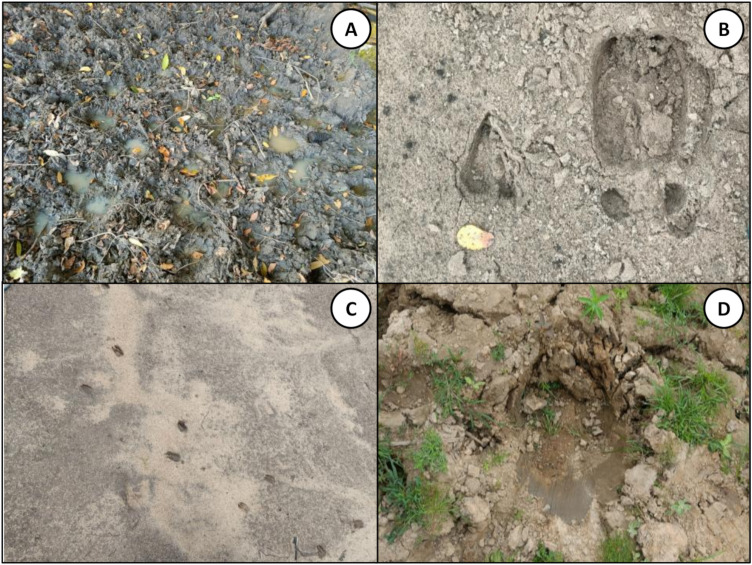
Examples of animal prints that were recorded as detections during the radial surveys of human, livestock, wildlife activity and land use. **A**: A collection of cattle hoofprints surrounding a pool. **B**: Hartebeest and a buffalo print in sand. **C**: Common duiker prints in sand. **D**: A hippo print on the riverbank.

Larval occupancy surveys were complemented by a survey for tracks (Fig 2), spoor or any other signs of activity by humans, livestock or wildlife around each aquatic habitat and *en route* between them [66,67,70]. This allowed the presence of larvae from any specific taxon found in individual aquatic habitats to be statistically associated with the frequencies of activity for each of the different mammalian species that were recorded around those water bodies at the time they were collected.

### Additional environmental variables

Before performing the statistical analyses, additional variables were added to the datasets to account for further spatial and environmental attributes of each camp location.

Ecosystem integrity was quantified using a subjective natural ecosystem integrity index (SNEII) based on a consensus of investigator impressions [67]. A list of the subjective ecosystem integrity scores for each camp is provided in Table S4.1 in S4 Appendix.

The variable *distance inside NNP and away from human settlements* was created to examine the effect of moving further into or away from fully intact wild areas, and also the potential influence of natural mosquito dispersal. Of particular interest, was the possibility of dispersal from suitable *source populations* in environments, where the species readily proliferates, into unsuitable environments that act as *population sinks* [78,79]. These distance values for each camp were calculated by measuring the number of kilometres from the camp coordinates to the nearest point on the NNP park boundary line (Fig 1), using QGIS Version 3.30.2. Camps that were located outside the NNP boundary were assigned a negative value, whereas camps that were located inside the NNP boundary were assigned a positive value. Camp 40 required particular consideration because, despite being one of the furthest camps inside the park, located approximately 40 km from the nearest point on the NNP boundary, it was also located just 16 km from the Julius Nyerere Hydropower Plant construction site (Fig 1). At the time, there was a large resident human settlement established there, potentially providing a more suitable environment for anthropophilic mosquito populations than would otherwise be the case so far inside such a large conserved area. Therefore, in order to improve the fit of relevant models, the value for this particular camp was adjusted to the distance from this known permanent human settlement inside the park rather than the distance to the nearest NNP boundary.

There were three readily distinguished historical landcover types found across the study area; evergreen groundwater forest, moist deciduous miombo woodland savanna, and dry acacia savanna. The predominant landcover type was assigned for each camp by the investigators, based on their recollections, the historical knowledge of Village Game Scouts regarding previous vegetation cover in heavily domesticated areas, and satellite imagery available through Google Earth^®^.

### Statistical analyses

The following statistical analyses were all completed using R Version 4.3.1

#### Aquatic habitat characteristics and occupancy by *Anopheles gambiae* complex larvae.

The dependence of the proportion of aquatic habitats occupied by *An. gambiae* complex upon various biotic and abiotic factors (S5 Data) were examined by fitting logistic generalized linear mixed models (GLMMs) that accounted for geographic variation associated with camp location and temporal autocorrelation with weekly time increments (S6 Appendix). The final best fit multivariate model was identified using a forward-step selection process (S6 Appendix).

#### Testing for evidence of competitive co-existence of *Anopheles arabiensis* and *Anopheles quadriannulatus.*

The Pearson’s correlation coefficient was used to identify any linear relationship between the absolute numbers of *An. arabiensis* and *An. quadriannulatus* within individual aquatic habitats.

#### Effects of environmental variables on *Anopheles gambiae* complex species composition.

Logistic GLMMs were fitted to the aggregated round 4 data (S7 Data) detailing how many PCR-identified *An. arabiensis* and *An. quadrianulatus* were collected, to investigate the effects that distance, ecosystem integrity and landcover may have had on *An. gambiae* complex population composition. These logistic GLMMS of the proportions of larvae identified as *An. arabiensis* rather than *An. quadriannulatus* accounted for habitat effects nested within camp location effects. An objective forward stepwise selection process was used to identify the final best-fit multivariate model (S6 Appendix).

#### Associations between *Anopheles gambiae* complex species composition and the availabilities of various potential hosts.

Similarly, another forward stepwise selection process to identify a best fit logistic GLMM was applied to examine possible influences of varying availabilities of different types of mammals as potential blood sources, recorded in terms of the total numbers of detections of activity for each species, upon sibling species composition of *An. gambiae* complex in larval samples (S6 Appendix). A Pearson’s correlation test confirmed close covariance between detected activity levels of humans and cattle (S9 Fig), so estimates for these two host species were merged as a new variable that summed the total numbers of detections of humans and the number of total detections of cattle herds.

Note, however, that the spatial and geographic parameters described in the section immediately above were not considered for inclusion in these GLMMs, based on a logical conceptual framework regarding expected distal associations versus underlying proximal causality (S8 Appendix). Similarly, lions, hyena, and leopard were all considered to be closely associated with wild herbivore populations, albeit in complex ways, and to contribute negligibly to overall mammalian biomass, so they were not considered for inclusion in the multivariate models (S8 Appendix).

### Ethical considerations

The procedures for this study were reviewed and approved by the Institutional Review Board of the Ifakara Health Institute (Ref. IHI/IRB/5–2021), the National Research Ethics Committee of the National Medical Research Institute (Ref. NIMR/HQ/R.8a/Vol. IX/3719) and the Tanzania Wildlife Research Institute (Ref. AB.235/325/01/37) in the United Republic of Tanzania, as well as the Animal Experimentation Ethics Committee of University College Cork (21/001). Permission to publish this study was kindly provided by Director General of the National Institute for Medical Research of the United Republic of Tanzania.

## Results

### Aquatic habitat occupancy by *Anopheles gambiae* complex larvae

**Fig 3:**
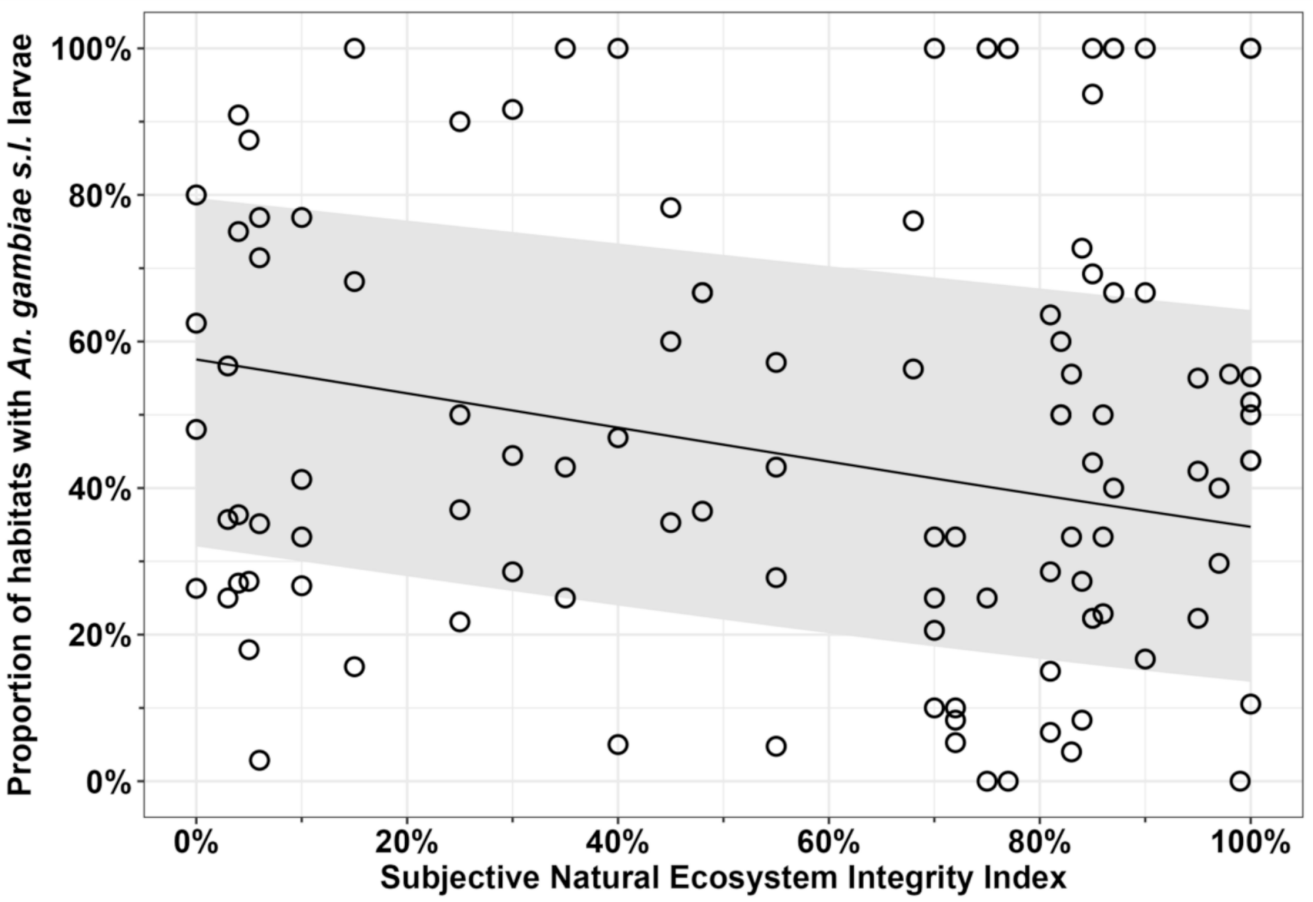
The proportion of habitats occupied by *An. gambiae* complex larvae for each survey conducted over four rounds plotted as a function of ecosystem integrity. The estimated mean trend with 95% confidence intervals reflects the predicted values for each surveyed camp location based on a fitted GLMM that is identical to the multivariate model detailed in S10.2 in S10 Appendix, except that all fixed effects other than the subjective natural ecosystem integrity index (SNEII; S4 Appendix and [66,67]) were instead treated as random effects, so that the predictions reflect the absolute means rather than the means for the reference values of each variable.

As detailed in Table S10.1 of S10 Appendix, larvae from the genus *Anopheles* were found in 1,058 (54.4%) of all 1,944 surveyed aquatic habitats. Of these, 765 (72.3%) included *An. gambiae* complex larvae, representing an overall occupancy rate of 39.4%. Prevalence of *An. gambiae s.l.* across larval habitats declined only slightly with ecosystem integrity (Fig 3 and Table S10.2 in S10 Appendix), with mean occupancy rates exceeding 25% even in the most intact natural environments (Fig 3), all of which were deep inside NNP (Table S4.1 in S4 Appendix).

### Competitive co-existence *Anopheles arabiensis* and *Anopheles quadriannulatus*

**Fig 4:**
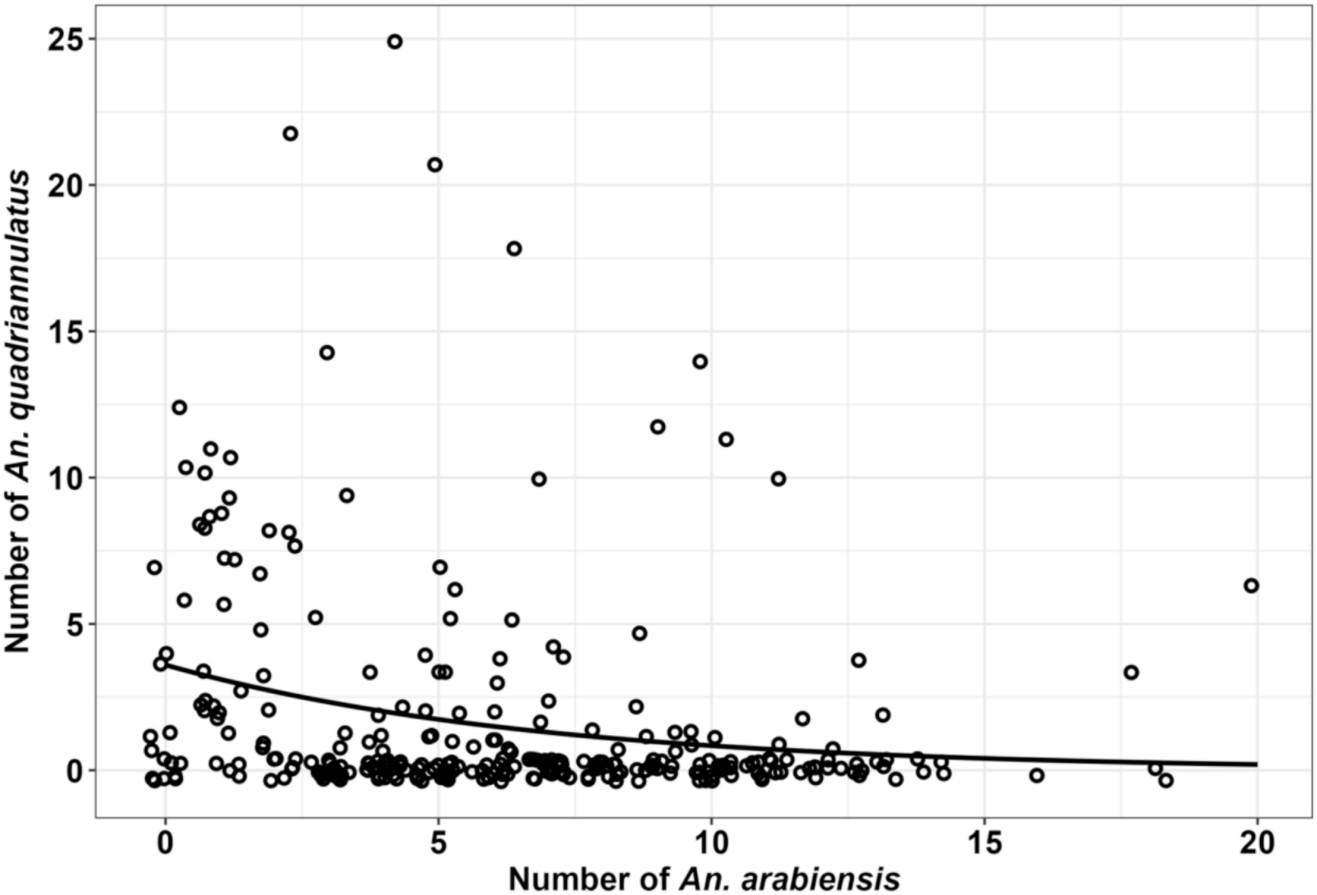
The number of *An. arabiensis* larvae per habitat plotted against the number of *An. quadriannulatus* larvae per habitat, as determined during the full fourth round of surveys.

Initial PCR results from amplified *An. gambiae* complex specimens demonstrated that these *An. gambiae* complex populations were not only comprised of *An. arabiensis* but also large numbers of *An. quadriannulatus*. These two sibling species were often found co-existing alongside each other in the same habitats on the same day (Fig 4).

Although variable species composition within the *An. gambiae* complex could have been explained by independently varying densities of the two sibling species found within the study area, this did not appear to be the case: As illustrated in Fig 4, the absolute numbers of *An. arabiensis* identified at each habitat were negatively correlated (*ρ* = −0.31, p = < 0.0001) with the absolute numbers of *An. quadriannulatus* found. In simple terms, wherever there were more *An. arabiensis,* there tended to be less *An. quadriannulatus*, and *vice versa*, confirming that these two species appear to compete with each other in the strict sense (S11 Text) to at least some extent.

### Effects of distance, ecosystem integrity and landcover on *Anopheles gambiae* complex species composition

Preliminary PCR results also demonstrated that while only *An. arabiensis* were found in fully domesticated and highly encroached locations, *An. quadriannulatus* were found around camps with largely undisturbed natural land cover, with SNEII scores of 50% or higher, and were most common in the best conserved locations (S12 Fig), suggesting that this non-vector utilizes wild animal hosts for blood feeding. The relative abundance of *An. arabiensis* declined as SNEII increased and the numbers of cattle herds detected became fewer (S12 Fig). This suggests that the outcome of its competitive interactions with *An. quadriannulatus* may be influenced by the availability of this known preferred host for the former species [16,80,81]. However, further investigation was required to find out whether *An. quadriannulatus* completely replaces *An. arabiensis* deeper inside NNP, even further away from humans and livestock.

**Fig 5:**
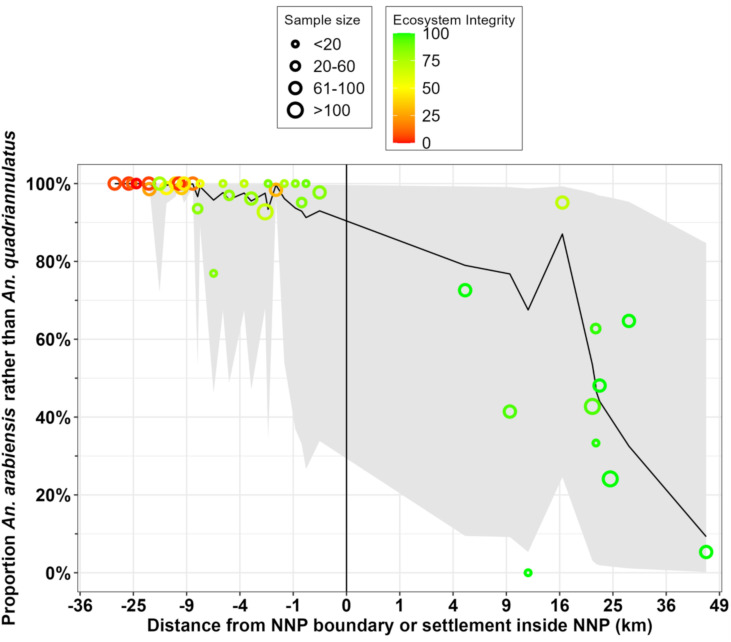
The proportion of field-identified *An. gambiae* complex collected and preserved *in situ* in the fourth round that were identified as *An. arabiensis* rather than *An. quadriannulatus* by PCR [77] plotted against distance to the nearest boundary of Nyerere National Park (NNP), with locations outside the park indicated by negative values. Symbol size indicates the number of specimens identified, collected and successfully amplified at that location, while colour indicates scores for the Subjective Natural Ecosystem Integrity Index (SNEII; S4 Appendix and [66,67]). The predicted mean values and their 95% confidence intervals for each surveyed camp location are respectively plotted as a black line and grey ribbon, both of which were calculated based on the estimated intercept and the fixed effects of distance and SNEII from the final multivariate model detailed in Table 1.

Distance from the NNP boundary or nearest human settlement inside the park, SNEII, and historical landcover were all found to be determinants of the sibling species composition of the *An. gambiae* complex by univariate analysis (Table 1). However, historical landcover type was excluded from the final multivariate model (Table 1). The best fit GLMM of these spatial and environmental covariates indicate that larval habitats located closer to human settlement and at locations with low ecosystem integrity scores, have higher proportions of *An. arabiensis* rather than *An. quadriannulatus* (Table 1, Fig 5). As larval habitats are located further away from the boundary and in areas of improved ecosystem integrity, the inverse effect occurs so that the odds of *An. gambiae* complex larvae amplifying as *An. arabiensis* decline by 59% for every 10 km further past the boundary and by 5% for every percentage point increase in the SNEII (Table 1, Fig 5). This indicates that *An. quadriannulatus* may have a competitive advantage in fully intact natural ecosystems far away from human settlements and their associated cattle herds.

**Table 1:** Univariate and multivariate outputs of generalized linear mixed models (GLMMs) for the effects of spatial and geographic attributes on the proportion of *An. arabiensis* rather than *An. quadriannulatus.* All model outputs were fitted to a binomial distribution with logit link function. A random effect that nested breeding site identification number within camp was included to account for variation between and covariance within larval populations. Statistically significant effects are highlighted in bold.

	Univariate			Multivariate		
Fixed Effects	OR [95% CI]	z	P	Proportion^a^ or OR^b^ [95% CI]	z	P
*Intercept*	NA	NA	NA	0.99 [0.99, 0.99]^a^	6.22	<<0.0001
Geographic and ecological attributes						
*Distance*	**0.21 [0.20, 0.21]** ^**c**^	**−8.37**	**<<0.0001**	**0.41 [0.40, 0.43]** ^**c**^	**−4.81**	**<0.0001** ^**e**^
*SNEII*	**0.97 [0.88, 0.93]** ^**d**^	**−6.56**	**<<0.0001**	**0.95 [0.92, 0.98]** ^**d**^	**−3.66**	**0.0002** ^**e**^
*Land cover*						
Miombo Woodland	1.00	NA	NA	1.00	NA	NA
Groundwater Forest	0.87 [0.02, 44.00]	−0.08	0.9332	1.03 [0.17, 6.27]^b^	0.03	0.974^f^
Acacia Savanna	**0.01 [<0.01, 0.12]**	**−3.75**	**0.0002**	1.67 [0.52, 5.33]^b^	0.86	0.388^f^
						
Random Effects				σ		
*Breeding site identification number/Camp number*				0.835		
*Camp number*				0.854		

^a^Proportion identified as *An. arabiensis* rather than *An. quadriannulatus* under reference conditions for all fixed effects.

^b^OR; Odds ratio.

^c^Odds ratio for every 10 km further towards the NNP boundary or past it and deeper into the park

^d^Odds ratio for each percentage point increase along the subjective natural ecosystem integrity index (SNEII).

^e^As estimated from the final best fit GLMM where the effect was included based on a significant value (P ≤ 0.05) and contributed to a lower AIC score.

^f^As estimated from the point at which the effect was no longer significant (P > 0.05) in the multivariate model fit and increased the AIC score, and was therefore excluded from the final model.

^σ^standard deviation

95% CI; The 95% confidence interval estimated for the proportion or OR.

NA; Not applicable because several different intercepts were estimated for more than one fitted univariate model, or not applicable to the reference group. SNEII: Subjective natural ecosystem integrity index (S4 Appendix and [66,67])

**Fig 6:**
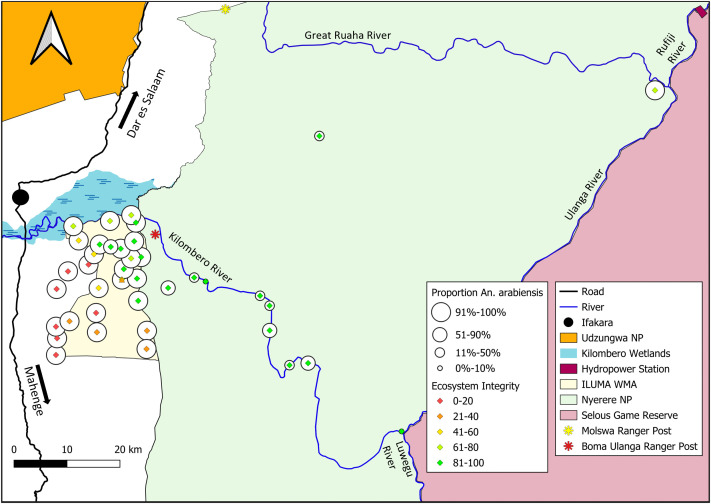
Map of ILUMA WMA and NNP illustrating how the proportion of *An. arabiensis* versus *An. quadriannulatus* varies geographically with respect to distance inside the park and away from human settlements, as well as the subjective natural ecosystem integrity (SNEII; S4 Appendix and [66,67]) at each camp location (Fig 1). This map was produced with QGIS® version 3.30.2 open-source software, using base maps obtained from OpenStreetMap® under the Open Database License.

The map in Fig 6 clearly illustrates how *An. arabiensis* dominated fully domesticated habitats around villages and all throughout ILUMA WMA, even though very low proportions (<7%) of *An. quadriannulatus* occurred in its best conserved areas, generally along the NNP boundary. A conspicuous change in species composition was observed at locations inside NNP (Figs 5 and 6), all of which were at least 5 km from the nearest boundary of NNP itself or that of an adjoining part of the Selous Game Reserve or Mikumi National Park. The SNEII reaches its maximum value at most camps inside NNP (Table S4.1 in S4 Appendix) and most of these had much higher proportions of *An. quadriannulatus* than any other camp in ILUMA or among the neighbouring villages to the west (Figs 5 and 6). Although the proportions of *An. arabiensis* generally declined with increasing distance inside NNP and away from substantial permanent human settlements (Figs 5 and 6), that geographic trend was remarkably erratic with lots of variability and wide confidence intervals (Fig 5), indicating considerable uncertainty regarding predictions of the fitted model presented in Table 1.

A clear outlier deep within NNP (Camp 40) represents a useful exception that proves the rule that *An. quadriannulatus* dominates the wildest areas because it had the highest proportions of *An. arabiensis* (95%) and the lowest SNEII score inside the national park (Fig 6, Table S4.1 in S4 Appendix). This camp was located approximately 16 km from the Julius Nyerere Hydropower Plant and an associated large human settlement within the national park. Indeed, the surveyed area around this camp had been exposed to intensive human activities, most notably considerable deforestation, over the previous two years, suggesting that this population of *An. arabiensis* may have been able to readily access human blood meals.

In contrast, camp 38 was located 47 km from the nearest boundary of NNP other than its border with the adjoining Selous Game Reserve (Fig 1), representing the furthest surveyed camp away from major human settlements. At this exceptionally well conserved location, only 5 of 76 (6%) of identified specimens were *An. arabiensis* (Figs 5 and 6), indicating a clear competitive advantage of *An. quadriannulatus* in this wild area. Nevertheless, it is notable that even in this remote and well conserved location, *An. arabiensis* persisted in small numbers. Indeed, camp number 30, which had a very small sample size (n = 3), was the only camp where were no *An. arabiensis* were identified (Figs 5 and 6). Overall, it seems that this species was ubiquitous across all surveyed parts of this very well protected park, and this interpretation is further supported by complementary surveys of adult mosquitoes across the same study area [82]. Notably, even the purposive impromptu survey carried out at *Mseguni*, which the escorting park rangers considered completely devoid of other humans or livestock, yielded 61 *An. arabiensis* along with 35 *An. quadriannulatus*.

### Associations between *Anopheles gambiae* complex species composition and potential host availability

The predicted means calculated from the GLMM detailed in Table 1 have wide confidence intervals (Fig 5), suggesting that the notable variability of *An. gambiae* complex species composition within the park may arise from heterogeneities in more specific factors other than distance and ecosystem integrity, such as blood resource availability and distribution. The following analysis was therefore undertaken to determine whether such variability in the competitive balance between *An. arabiensis* and *An. quadriannulatus* could be attributed to surveyable indicators of the activities of all the various mammalian species present in the study area, including humans and cattle.

The best fit model from multivariate analysis indicates that cattle, humans, impala, warthog and perhaps bushpig are all mammalian species that appear to influence the competitive balance between *An. arabiensis* and *An. quadriannulatus* (Table 2). Hippos and waterbuck appeared to be significant covariates in the univariate analysis but were later excluded from the multivariate models once impala was accounted for, indicating covariance between these three species, all of which were detected most frequently around water bodies inside NNP. Similarly, common duiker was also excluded from the multivariate model once warthog had been added. Slender mongoose and baboons were also dropped from the multivariate analysis for similar statistical reasons, suggesting that their geographical distribution covaried with that of the species retained within the model. Bushpig, which is a nocturnal near-relative of the warthog, was almost also retained in the final model, because it approached significance as a predictor of *An. gambiae* population composition (Table 2) and improved goodness of fit but only approached being significantly so (AIC = 496.7 versus 497.5, P = 0.0945).

**Table 2:** Univariate and multivariate outputs of generalised linear mixed models (GLMMs) of the effects of recorded detections of individual animal species on the proportion of *An. arabiensis* rather than *An. quadriannulatus*. All models were fitted to a binomial distribution with logit link function to the dependent variable. A random effect comprised of breeding site identification number nested within camp number was included to account for variation between and covariance within larval populations at individual waterbodies. Statistically significant effects are highlighted in bold.

	Univariate			Multivariate		
Fixed Effects	OR [95% CI]	z	P	Proportion^a^ or OR^b^ [95% CI]	z	P
						
*Intercept*	NA	NA	NA	0.98 [0.97, 0.99]^a^	8.82	<<0.0001
						
*Humans and Cattle Herds*	**717 [**57, **9107]**	**5.07**	**<<0.0001**	**29.1 [3.7, 226.6]** ^**b**^	**3.22**	**0.0013** ^**c**^
						
*Herbivores*						
Hippo	**0.10 [0.04, 0.22]**	**−5.78**	**<<0.0001**	0.46 [0.10, 2.21]^b^	−1.81	0.0701^d^
Impala	**0.11 [0.05, 0.24]**	**−5.56**	**<<0.0001**	**0.25 [0.15, 0.43]** ^**b**^	**−4.91**	**<0.0001** ^**c**^
Waterbuck	**0.21 [0.08, 0.56]**	**−3.15**	**0.0019**	1.04 [0.58, 1.89]^b^	0.14	0.8853^d^
Warthog	**0.29 [0.37, 2.73]**	**−2.39**	**0.0160**	**0.49 [0.32, 0.77]** ^**b**^	**−3.12**	**0.0019** ^**c**^
Bushpig	**0.26 [0.09, 0.80]**	**−2.33**	**0.0197**	0.62 [0.37, 1.06]	−1.75	0.0804 ^d^
Common Duiker	**5.31 [1.26, 22.9]**	**2.28**	**0.0225**	1.3 [0.56, 3.03]^b^	0.62	0.5354 ^d^
Eland	0.44 [0.15, 1.35]	−1.43	0.1530	NE	NE	NE
Zebra	2.39 [0.69, 8.33]	1.37	0.1700	NE	NE	NE
Buffalo	0.51 [0.17, 1.57]	−1.17	0.2420	NE	NE	NE
Elephant	0.59 [0.17, 1.71]	−1.05	0.2960	NE	NE	NE
Hartebeest	0.64 [0.19, 2.20]	−0.71	0.4790	NE	NE	NE
Bushbuck	0.77 [0.24, 2.40]	−0.46	0.6460	NE	NE	NE
Reedbuck	0.82 [0.39, 2.62]	0.35	0.729	NE	NE	NE
Suni	1.23 [0.33, 4.60]	0.30	0.7620	NE	NE	NE
Sable	0.84 [0.24, 2.93]	−0.27	0.7900	NE	NE	NE
Dik-dik	0.92 [0.26, 3.38]	−0.11	0.9120	NE	NE	NE
*Large Carnivores*						
Hyena	**0.18 [0.07, 0.48]**	**−3.45**	**0.0006**	NE	NE	NE
Leopard	**0.31 [0.12, 0.85]**	**−2.29**	**0.0222**	NE	NE	NE
Lion	0.53 [0.18, 1.54]	−1.17	0.2410	NE	NE	NE
*Small Carnivores*						
Slender mongoose	**0.20 [0.07, 0.61]**	**−2.82**	**0.0047**	0.96 [0.47, 1.95]^b^	−0.11	0.9100^d^
Civet	0.48 [0.16, 1.43]	−1.32	0.1880	NE	NE	NE
African wildcat	0.47 [0.16, 1.44]	−1.32	0.1860	NE	NE	NE
Water mongoose	1.61 [0.53, 4.88]	0.822	0.4110	NE	NE	NE
Genet	0.48 [0.21, 1.84]	−0.71	0.4800	NE	NE	NE
*Primates and Prosimians*						
Baboon	**0.24 [0.09, 0.67]**	**−2.72**	**0.0066**	0.75 [0.45, 1.26]^b^	−1.10	0.27357^d^
Vervet monkey	0.24 [0.75, 5.76]	0.88	0.3790	NE	NE	NE
Blue monkey	1.28 [0.40, 4.03]	0.42	0.6700	NE	NE	NE
Greater Galago	0.84 [0.29, 2.41]	−0.33	0.7400	NE	NE	NE
*Macroscelidea*						
Sengi	0.87 [0.29. 2.60]	−0.24	0.8070	NE	NE	NE
*Tubulidentata*						
Aardvark	0.67 [0.20, 2.41]	−0.60	0.544	NE	NE	NE
Random Effects				σ		
*Breeding site identification number nested within camp number*				0.868		
*Camp number*				1.078		

^a^Proportion identified as *An. arabiensis* rather than *An. quadriannulatus* based on the estimated intercept of the model.

^b^OR; Odds ratio per standard deviation increase from the mean.

^c^As estimated from the final best fit GLMM where the effect was included based on a significant value (P ≤ 0.05) and contributed to a lower AIC score.

^d^As estimated from the point at which the effect was no longer significant (P > 0.05) in the multivariate model fit and increased the AIC score, so it was excluded from the final model.

95% CI; The 95% confidence interval estimated for the proportion or OR.

NA; Not applicable because several different intercepts estimated for more than one fitted univariate model, or not applicable to the reference group.

NE; not estimated for variables that were not significant in the univariate analysis or large carnivores that were significant in the univariate analyses but excluded from the multivariate model on principle because they were considered to covary with herbivore abundance and represent negligible biomass in their own right (S8 Appendix).

σ; standard deviation

**Fig 7:**
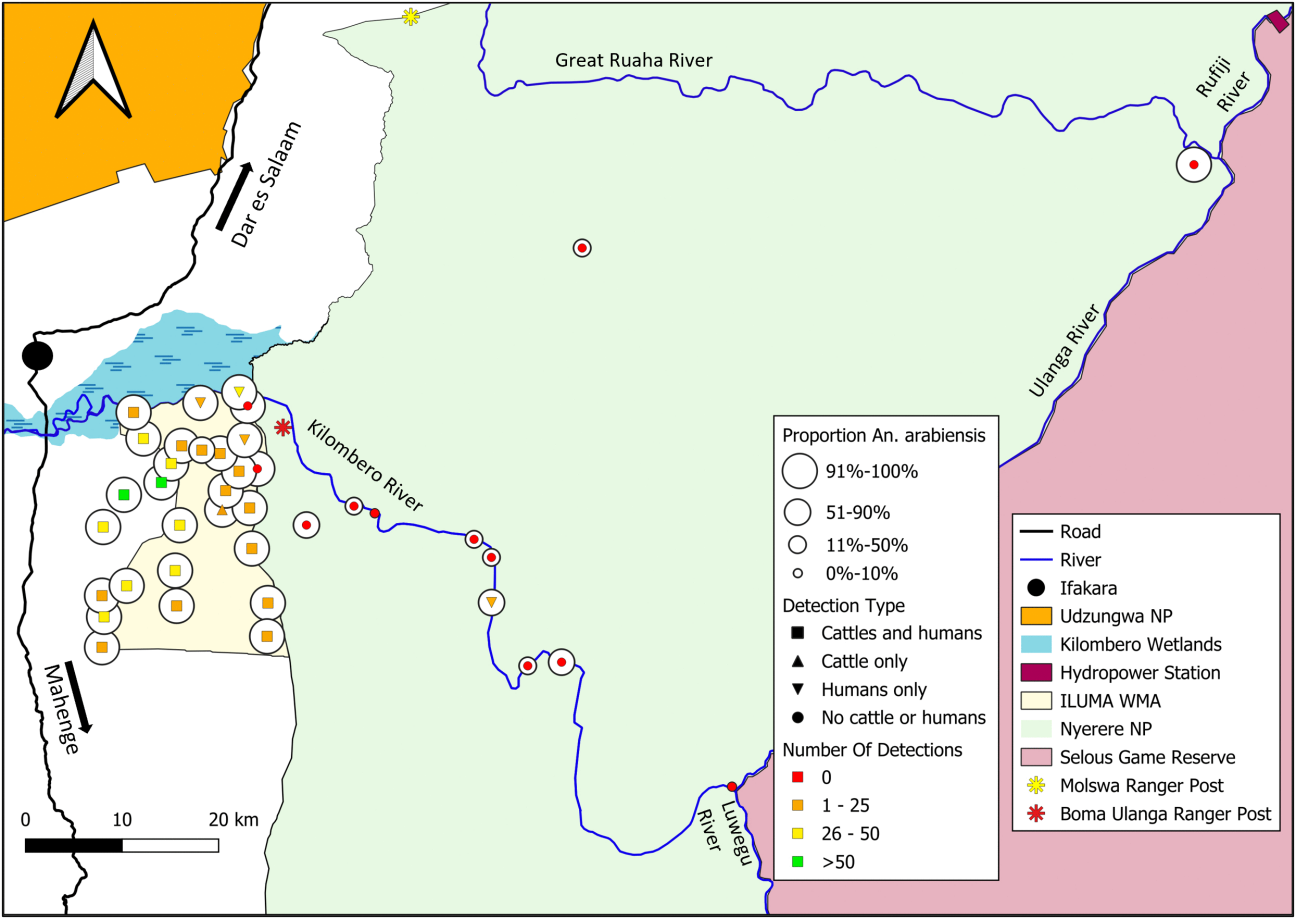
Map of ILUMA WMA and NNP illustrating how the proportion of *An. arabiensis* versus *An. quadriannulatus* varies geographically with respect to the number of times that activities of cattle and/or humans were detected at each camp (Fig 1). This map was produced with QGIS® version 3.30.2 open-source software, using base maps obtained from OpenStreetMap® under the Open Database License.

Humans and cattle had the greatest effect on the relative abundances of *An. arabiensis* and *An. quadriannulatus* collected from larval habitats (Table 2). Consistent with the initial descriptive observations (S12 Figure), such a high odds ratio indicates that where cattle or humans are present, populations of *An. gambiae* complex are overwhelmingly dominated by *An. arabiensis*. When their preferred blood sources become scarce or completely absent, however, proportions of *An. arabiensis* decline, with this species seemingly losing its competitive advantage. Detections of humans or cattle were most frequently recorded in fully domesticated habitats, where the proportions of *An. arabiensis* consistently exceeded 91% (Fig 7). The highest number of detections of humans and cattle were in the villages outside the ILUMA western boundary, and these two host species were less frequently detected around larval habitats inside ILUMA (Fig 7). Nevertheless, humans and cattle were present, even if only in small numbers, across most of the WMA, including places that were only a kilometre from the NNP boundary (Fig 7). Indeed, only two camps inside ILUMA WMA, (camps 9 and 13), appeared devoid of these hosts (Fig 7). However, given the extent of human and livestock encroachment at the surrounding camps, the high proportions of *An. arabiensis* found at these locations were likely within flying distance of the nearest human or cow.

Only one camp inside NNP (Camp 33), exhibited any signs of the presence of people (a few tracks of one or two fish poachers) and it is notable that it had higher proportions of *An. arabiensis* than most other camps inside NNP (Fig 7). This indicates that even a small number of humans may be enough to give *An. arabiensis* an advantage in a competitive relationship that they appear to dominate when allowed to.

Although no detections of humans or cattle were made at camp 40, which had a high proportion of *An. arabiensis* despite being located 16 km away from the nearest large permanent human settlement (Fig 5), this does not necessarily suggest long range dispersal by this species. The area around this camp was observed to be quite degraded and ongoing activities of humans and/or livestock may have occurred nearby but nevertheless beyond the reach of the surveys: While the two large rivers that converge nearby acted as a natural barrier that constrained the human survey team, ovipositing mosquitoes could have easily flown across from the unsurveyed opposite banks to the north and east.

**Fig 8:**
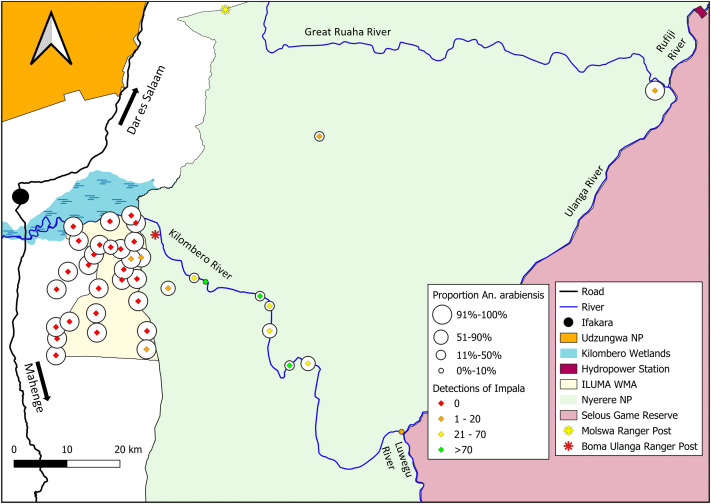
Map of ILUMA WMA and NNP illustrating how the proportion of *An. arabiensis* versus *An. quadriannulatus* varies geographically with respect to the number of times that activities of impala were detected at each camp (Fig 1). This map was produced with QGIS® version 3.30.2 open-source software, using base maps obtained from OpenStreetMap® under the Open Database License.

The relative abundance of *An. arabiensis* was strongly and negatively associated with impala activity (Table 2). Impala were the predominant and most abundant antelope recorded inside NNP, particularly at camps along the Kilombero river where *An. quadriannulatus* were, on average, at least as common as *An. arabiensis* (Fig 8). This suggests that this common antelope of dry savanna mosaic habitats [83] may well be a preferred blood source for *An. quadriannulatus*.

**Fig 9:**
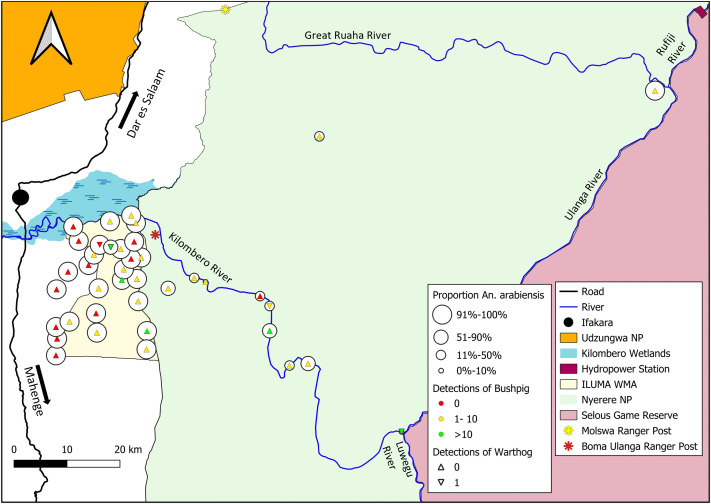
Map of ILUMA WMA and NNP illustrating how the proportion of *An. arabiensis* versus *An. quadriannulatus* varied geographically with respect to the number of times activities of warthog and bushpig were detected at each camp (Fig 1). This map was produced with QGIS® version 3.30.2 open source software, using base maps obtained from OpenStreetMap® under the Open Database License.

**Fig 10:**
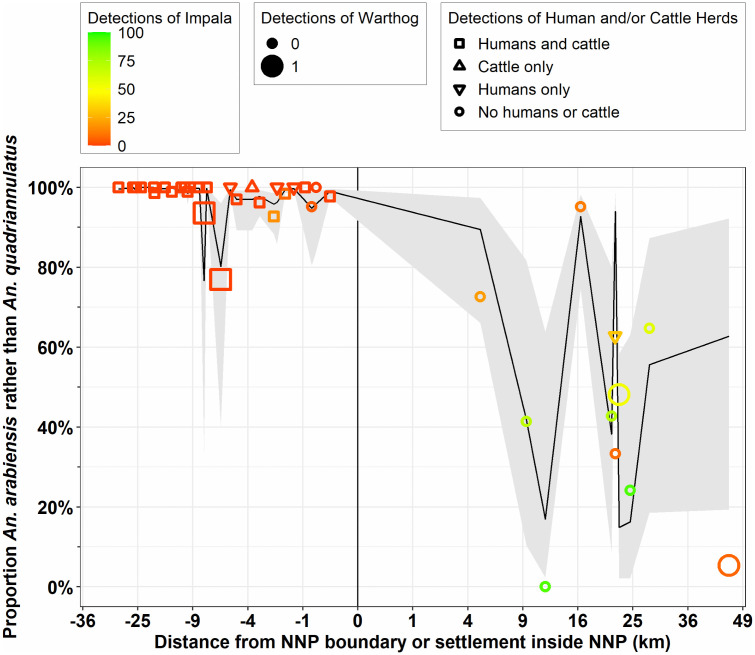
The proportions of field-identified *An. gambiae* complex specimens collected and preserved *in situ* during the fourth survey round that were identified as *An. arabiensis* rather than *An. quadriannulatus* by PCR [77] plotted against distance to the nearest boundary of Nyerere National Park (NNP) and away from human settlements, with locations outside the park indicated by negative values. Symbol colour indicates the number of impala detections, symbol size indicates the number of warthog detections and symbol shape indicates whether humans, cattle, both or neither were detected. The predicted mean values and their 95% confidence intervals for each surveyed camp location are respectively plotted as a black line and grey ribbon, both of which were calculated based on the total number of detections for cattle and humans, impala and warthog and the generalized linear mixed model (GLMM) presented in Table 2.

Table 2 details how the relative abundance of *An. arabiensis* also seemed to be negatively associated with signs of activity by warthog, and possibly also bushpig*,* despite both suid species being detected far less frequently than either impala, humans or cattle (Figs 9 and 10). This suggests that warthog, and perhaps it’s close relative the bushpig, may provide further preferred blood sources for *An. quadriannulatus*. Both locations within NNP where warthog were detected had high proportions of *An. quadriannulatus* (52% (41/82) at camp 34 and 95% (71/75) at camp 38) and both locations in ILUMA where warthog were detected were also among the minority of camps where *An. quadriannulatus* were found (32% (9/28)). Bushpig was almost also included in the final model (Table 2), suggesting that this more abundant and widely distributed wild suid might also represent a preferred host for *An. quadriannulatus*. Indeed, eight of the nine locations within ILUMA where *An. quadriannulatus* were found also had detectable populations of bushpig (6), warthog (1) or both (1) (Fig 9).

The estimated effect size for combined detections of cattle and humans on the relative abundance of *An. arabiensis* is much greater than the effect sizes estimated for impala, warthog or, tentatively, bushpig (Table 2), suggesting that *An. arabiensis* have a very strong competitive advantage over *An. quadriannulatus* when their preferred blood sources (cattle or humans) are present at even low densities, only allowing *An. quadriannulatus* to compete with it in wild areas far away from human settlement and livestock.

Taken at face value in mathematical terms, such a considerable effect size for humans and cattle (Table 2) suggests *An. arabiensis* might be expected to disappear in the complete absence of these two known preferred hosts. Instead, however, it persists at relative abundances that are generally readily detectable and usually exceed 20% deep inside NNP (Fig 10). Even at the furthest systematically surveyed location from any substantive human settlement (camp 38), five *An. arabiensis* larvae were identified (Fig 10) and it is notable that each was collected from a different aquatic habitat. It is highly unlikely that these five specimens collected from five separate habitats could have hatched from eggs laid by a single common mother that had fed on a person or cow outside the park and then flown almost 50 km before ovipositing. It therefore seems far more likely that these specimens represent a self-sustaining wild population that feeds on one or more alternative wild hosts that have not been captured by the best fit GLMM in Table 2.

Reassuringly, the fluctuations of relative abundance between these two mosquito species across a gradient of fully domesticated to essentially intact natural ecosystems, appears to be better explained by data from surveys of mammalian activity (Fig 10, Table 2), than by broader geographic parameters, namely distance and the SNEII (Fig 5, Table 1). Although the final multivariate host species model presented in Table 2 has a similar goodness-of-fit to that based on geographic and ecological variables in table 1 (AIC = 497.5 versus 490.3, respectively, P = 1.00), it more effectively captures the considerable variability between camps within NNP and yields far narrower confidence intervals (Fig 10 versus Fig 5). The one major outlier is also informative: The model prediction also failed to capture the low proportions of *An. arabiensis* and high proportions of *An. quadriannulatus* recorded deep inside NNP at camp 38, where impala and warthog were barely detected (Figs 8 and 9) but bushpig activity was recorded 11 times, placing it in the top 12^th^ percentile for the latter species. This observation seems consistent with the interpretation that *An. quadriannulatus* may also feed on bushpig, as suggested by the fact that it was only barely excluded from the final best-fit model in table 2 on marginal statistical grounds. Reassuringly, the only ILUMA location with *An. quadriannulatus* where neither suid was found had by far the highest levels of impala activity (17 detections at camp 10), out of only three locations where this antelope was found within the WMA.

## Discussion

Although no specific mammal species could be identified as a potential alternative blood source to cattle or humans that *An. arabiensis* are known to prefer, the results presented herein clearly demonstrate a competitive relationship between *An. arabiensis* and *An. quadriannulatus* where these two sibling species coexist within well conserved natural ecosystems. While *An. arabiensis* is a stereotypical vector of residual malaria transmission [7,11,13,22], *An. quadriannulatus* is generally considered to be a non-vector [4,16,50]. This study implicates impala, warthog and possibly bushpig as likely preferred blood sources for the relatively harmless latter species, allowing it to dominate the more dangerous former sibling species in areas where these mammals occur. Having said that, the results presented also indicates that self-sustaining refuge populations of *An. arabiensis* nevertheless exist in even the most remote, well protected conservation areas where humans and livestock are essentially absent, where they presumably survive by feeding on one or more wildlife species that remain to be identified.

### Distribution of Anopheles quadriannulatus

At the outset of the study, *An. arabiensis* was thought to be the only persisting member of the *An. gambiae* complex in the Kilombero valley since the elimination of *An. gambiae s.s.* in this area by LLINs circa 2010 [8,10,65]. It was therefore assumed *a priori* that it would be the sole sibling species present, surviving in the wilderness by extending its ability to feed on cattle to wild bovids. Instead, the data revealed the presence of the much more zoophagic species *An. quadriannulatus* [4,16,50,51] in conserved wild areas. This observation was completely unanticipated given that only three specimens of this species had previously been identified over the long history of mosquito research in the Kilombero valley [84]. The remarkably strict zoophagy of this *An. quadriannulatus* population is clearly confirmed by the fact that similarly intensive parallel surveys of adult mosquitoes across the same sampling frame with human-baited traps yielded only 29 specimens of this species, contrasting starkly with the 3,658 *An. arabiensis* caught, even though the former dominated the latter in local larval populations at many of the wilder places sampled [85]. The sympatric populations of the two sibling species reported herein based on larval surveys are consistent with the historical literature across Africa, which indicates that *An. quadriannulatus* occurs in the same geographic regions as *An. arabiensis* [4], although the distribution of the former is much patchier than the latter [4,50].

In general, studies of *An. gambiae* complex focus on human exposure to the most important malaria vectors and therefore upon inhabited areas. Although behaviourally plastic malaria vectors like *An. arabiensis* feature strongly, this study illustrates clearly how more strictly zoophagic species like *An. quadriannulatus* may be underrepresented in such studies around towns, villages and farms. As demonstrated herein, finding self-sustaining populations of *An. quadriannulatus* may require sampling of conserved wilderness areas at least 10 km from the nearest human settlement. Indeed, this species may have a far wider distribution than previously appreciated because their apparent inability to compete with *An. arabiensis* wherever humans and cattle abound restricts them to remote, conserved wilderness areas that are rarely surveyed.

Furthermore, these observations perhaps shed light on the apparent scarcity of *An. quadriannulatus* across Africa over recent years when compared with historical records. When much of the classical literature on African *Anopheles* was written half a century ago [4,16], rural settlements across the continent were generally fewer, smaller and frequently surrounded by natural ecosystems with healthy wildlife populations. Today, the opposite is true: Increasingly, intact wilderness is found only inside protected areas that are surrounded by growing populations of humans and livestock. It is therefore plausible that the already relict range of *An. quadriannulatus* has contracted even more over recent decades.

### Competitive co-existence of *Anopheles arabiensis* and *Anopheles quadriannulatus* populations

The presence of *An. quadriannulatus* in varying proportions gave insights into the potential drivers of their ability to co-exist with *An. arabiensis* in fully conserved wilderness, despite their apparently competitive relationship (S11 Text). According to the classic monograph of Gillies and Coetzee [16], the range of aquatic habitats used by all the various freshwater species of the *An. gambiae* complex appear to be very similar, without any obvious differences between sibling species. While some distinctions in habitat use have been documented more recently between other members of the same complex, namely *An. gambiae s.s.* and *An. coluzzii*, these are nevertheless relatively subtle [86,87]. The co-occurrence of both *An. arabiensis* and *An. quadriannulatus* in the same aquatic habitats is therefore unsurprising [80,81,88,89].

Although competition at larval stages, and differences in their preferred environmental niches, may both contribute to the fluctuating species composition observed in this study, it is also important to consider the full mosquito life cycle because each life stage is interdependent. For example, competitive interactions at larval stages influence adult population density and fitness [90–95]. Conversely, however, larvae cannot occupy habitats without a female first successfully acquiring a blood meal and then finding and ovipositing in it.

It is therefore unsurprising that the competitive balance between *An. arabiensis* and *An. quadriannulatus* larvae within aquatic habitats (Fig 4), and between other pairs of species within the *An. gambiae* complex, are so clearly influenced by the availability of preferred blood hosts nearby [80,81] and by vector control measures that protect some of those hosts against attack but not others [8,9,10,90,96–101]. Indeed, such competitive displacement by closely related species may well help explain the remarkably swift and decisive elimination of highly human-specialized malaria vectors by LLINs or IRS on several occasions, in a rapid and decisive manner that cannot be explained with simple single-population models [10,102].

Although *An. arabiensis* seems to dominate *An. quadriannulatus* wherever it can access its known preferred hosts (cattle and humans) [18,19], the reverse does not appear to true: *An. quadriannulatus* does not completely displace *An. arabiensis* where its inferred preferred hosts (impala, warthog and possibly bushpig) are present, even when cattle and/or humans are absent. Instead, *An. quadriannulatus* merely supresses *An. arabiensis* abundance. Indeed, complementary surveys of adult mosquitoes confirm that *An. arabiensis* is ubiquitous across the study area, even including the wildest areas where people and livestock are essentially absent but impala, warthog and/or bushpig are relatively abundant [82,85]. It therefore seems likely that *An. arabiensis* can make use of blood from one or more wild mammals with sufficient frequency to persist even in the face of clear competition from *An. quadriannulatus* in the wildest areas furthest away from humans and cattle.

### Mammalian blood host availability and inferred host preferences of mosquitoes

Blood source utilisation patterns of *An. arabiensis* have been repeatedly characterized in diverse settings across Africa [7] but the blood feeding habits of *An. quadriannulatus* are far less well understood. The clear association of *An. arabiensis* with signs of cattle or human activity reported herein is consistent with the known host preference behaviours of this malaria vector [4,16–18,19]. The remarkably high intercept and strong effect size for detected activities of humans and cattle displayed in Table 2, is consistent with the results of fitting process-explicit models to bloodmeal identity data from pastoralist communities in northern Tanzania, demonstrating that these were the only two frequently used hosts for this mosquito species [19].

As *An. quadriannulatus* are generally thought to be of little relevance to malaria transmission [4,16,50], their blood feeding behaviours have rarely been studied and remain poorly understood. Where this vector occurs in domesticated areas, it predominantly feeds on cattle [4] but will also feed on humans in some settings [103–105]. Nevertheless, according to the classical literature*,* it is thought that *An. quadriannulatus* primarily survives on wild animals [4,16] and a parallel survey of adult mosquitoes overlaid upon this study confirms this particular population is comprehensively unresponsive to human hosts [85]. However, conclusive bloodmeal analysis for this species is almost entirely lacking, presumably because it is notoriously difficult to collect resting blood fed specimens of such zoophagic mosquito species that are usually also exophagic [106–111]. One study inside Mana Pools National Park, Zimbabwe [24], identified mixed blood-meals in a few specimens that included DNA from cattle and other unidentified hosts that were presumed to be wild ungulates. However, clear-cut evidence for a direct link between *An. quadriannulatus* and a specific wild host species has not yet been reported to our knowledge.

It is therefore encouraging that impala was indirectly identified herein as the most plausible and common blood source utilized by these *An. quadriannulatus* populations, an adaptation that might be attributed to the specific ecological characteristics of this antelope. Impala were the most frequently detected antelope in parts of NNP where *An. quadriannulatus* was most abundant and breeding herds of impala can comprise as many as 120 individuals [83]. In conserved habitats they find suitable, impala represent a considerable amount of mammalian biomass [83], an important factor determining the host choice of blood-seeking adult mosquitoes [112–116]. Furthermore, impala readily switch from grazing in the wet season to browsing in the dry season, enabling them to remain all year round within much smaller home ranges (often less than 1km^2^) than larger more specialised antelopes [83]. Furthermore, these compact home ranges are specifically established close to perennial surface water sources, so they can drink daily and browse on the trees, shrubs and bushes that grow around such waterholes and streambeds throughout the dry season [83]. Impala are also least active after dusk and spend most of this time lying down [83], coinciding with the peak crepuscular biting activity peaks that are generally associated with zoophagy in mosquitoes [7,13,21]. Impala may therefore represent an especially reliable, abundant and historically widespread blood resource that is often concentrated around waterbodies where emergent blood-seeking mosquitoes may breed, and therefore an astute choice for *An. quadriannulatus* to have evolved to specialise upon.

Host-specialised blood utilisation behaviours are very common among anopheline mosquitoes, occurring in 82% of all studied species [116,117]. Such specialisation to feed on only one or two vertebrate species is thought to have evolved through selection by several factors, notably the rates at which various potential host species are encountered [117]. For example, it has been shown that the speciation of the highly human-specialised *An. gambiae s.s.* arose with the arrival of Bantu agriculturists in the forest belt of central Africa [23]. Similarly, the unusually high historical abundance of impala across all the mixed acacia savannah habitat of eastern and southern Africa [83] may well have underpinned the evolution of *An. quadriannulatus* to specialise on this antelope, which remains widespread across protected areas today.

Nevertheless, it is important to also consider the limitations of associative observational studies when inferring preferred blood resources. For example, because other mammalian species were not detected as frequently inside NNP, the models detailed in Table 2 had less statistical power for evaluating the influence of these other potential blood host species. Correspondingly, a lack of other attributable influences may not necessarily imply that these numerous alternative mammals are not utilised as blood sources just as readily. It may well be the case that *An. quadriannulatus* simply feed flexibly and opportunistically upon whatever the most readily available hosts happen to be in any given locale, with impala being by far the most abundant in this case.

That said, the statistical inference that warthog, and possibly also bushpig, may represent additional potential preferred blood hosts provides encouraging evidence of the validity and power of the approach taken. This is because even the small number of detections of these two closely related suid species, when compared to many other wild animals, proved sufficient to give plausible evidence that *An. quadriannulatus* may well utilise them as blood sources. Like impala, both warthog and bushpig are social animals that live in family groups [83]. Like impala, both suids are sedentary, staying permanently within remarkably small home ranges with ready access to surface water all year round [83], thereby offering quite reliable feeding opportunities for mosquitoes.

While the survey methodology proved useful for inferring host species, the greatest overall limitation of this study is simply that it was purely observational in nature and can therefore only demonstrate plausible indirect associations rather than probable direct causality. In the absence of corroborating evidence from other sources, the implication of impala, warthog and perhaps bushpig as apparent blood sources for *An. quadriannulatus* could, of course, be mere artefacts that arose by random chance from spurious model fits. Further studies will be required to address this uncertainty, with more locations sampled deep inside wilderness areas, more than one cross-sectional survey round that distinguishes between larvae collected from different individual habitats, more nuanced regression methods and improved field techniques for collecting elusive blood fed mosquitoes resting outdoors. Having said that, it is encouraging that the overall confidence intervals for the predicted proportions of *An. gambiae s.l.* larvae identified as either of these two sibling species are far narrower when based on models of potential blood host abundance (Fig 10), rather than the geographic and ecological variables (Fig 5) that were assumed to be more indirectly associated with (S8 Appendix). This would appear to confirm the conceptual basis of these complementary analyses (S8 Appendix), with the latter assessing possible distal determinants of what blood sources were most readily available to mosquitoes in each location, while the former examined far more direct, proximal measures of potential host availability.

Furthermore, it is, of course, reasonable to question the reliability of the methodology used to survey the activities of humans, livestock and wild mammals, especially given the dense vegetation cover across much of the study area and the cryptic habits of many of the latter. However, practical experience during this study and detailed analysis of the animal activity survey data [66,67,70] both confirm that indirect observations of tracks, spoor and other signs were quite sensitive and reliable indicators, revealing the presence of almost every species envisaged at the outset, including 15 that were never directly sighted over the course of the two years. Although the activities of humans, livestock and wild animals were detected in several different ways, footprints were by far the most frequent indicator found around the perimeter of waterbodies, where the moist soil facilitated clear track indentations that could be confidently identified [66,70].

Despite its uncertainties, this novel methodology for associating the species composition in larval surveys with such indirect indicators of host availability has proven a simple, yet effective and powerful approach that implicated four different preferred hosts of the *An. gambiae* complex within the study area; two for *An. arabiensis* and two for *An. quadriannulatus.* Beyond this malaria-related example, this new approach to studying host preferences of adult mosquitoes, based on surveys of their larval habitats and the various mammals that frequent them, may have much broader applications for studying a wide range of vector borne zoonoses and emerging pathogens. Specifically, it may be deployed to fill a strategic methodological gap by allowing exploration of the adult feeding habits of zoophagic species, the vast majority of which are very poorly understood because they predominantly feed on wild hosts in expansive outdoor habitats and are consequently very difficult to capture or observe [106–110,118].

Fortunately, the larval stages of mosquitoes are confined to waterbodies and so are much easier to collect and identify than free-flying adults lacking interest in human bait hosts. Therefore, the survey approach described herein could be applied to explore the full diversity of mosquitoes found in such aquatic habitats and the broad spectrum of blood sources they are thought to specialize upon to establish their distinctive ecological niches [117,119]. Crucially, this approach may even be applied to very cryptic wild vertebrate hosts that are difficult to survey by direct observation: For example, the bushpig implicated herein as a possible blood source for *An. quadriannulatus* were never actually seen by the investigators during this study. By mapping out overlaps between mosquito species in terms of their preferred hosts in this way, it may well be possible to indirectly identify bridge vector species that could transfer pathogens from various wildlife species into livestock and/or humans.

That said, the existence of *An. arabiensis* deep inside NNP could not be explained by any of the variables retained in the final multivariate model in table 2, so it was not possible to address the original primary objective of the study, which was to associate wild populations of *An. arabiensis* with one or more hosts other than humans and cattle. Prior to data collection, it was hypothesised that perhaps African buffalo might provide a plausible blood source for wild type mosquitoes because of their close phylogenetic relationship with cattle [120], combined with their size and propensity to often form large herds [83]. This common wild bovine species has previously been suggested as a potential blood source for a wild population of *An. arabiensis* identified in Kruger National Park, South Africa, based on the observation that they frequented aquatic habitats that were occupied by larvae [25]. Furthermore, bloodmeal analysis from a study in Uganda identified African buffalo DNA in a blood-fed *An. gambiae* complex mosquito but unfortunately the specimen was not identified to sibling species level [121]. As the host association model in Table 2 did not provide any evidence that the abundance of African buffalo or any wild herbivore favoured *An. arabiensis*, questions remain regarding the ecological niche of this vector species outside of landscapes that are at least partially domesticated, with humans and/or cattle available to support its reproduction.

### Plausibility of self-sustaining *Anopheles arabiensis* refuge populations in remote, fully intact wilderness areas

Given that *An. arabiensis* appears so well adapted to dry environments [1–3,6,52,122], their presence deep inside NNP might possibly be explained by dispersal from surrounding domesticated lands. While, host-seeking adults from the *Anopheles gambiae* complex usually exhibit modest flight range of a few hundred metres to a few kilometres [16,72,123], they also sometimes disperse much further by exploiting high altitude winds [124–126]. Indeed, as no wild alternative hosts for *An. arabiensis* could be identified for this vector species inside fully intact, well protected wilderness areas (Table 2), it could be argued that these are *sink* populations [78,79] that may not be independently viable without sustained influx of already blood-fed adults from surrounding *source* populations [78,79] in domesticated villages. However, it is difficult to envisage how such long-range dispersal alone could sustain such readily detected densities of *An. arabiensis* so deep inside NNP for several reasons.

First, the reciprocal of this spatial distribution pattern for *An. arabiensis* was not observed: Despite the presence of abundant *An. quadriannulatus* inside NNP, no specimens of this species were found in any of the nearby villages or even across most of the ILUMA conservation area. Notably, all nine sampled locations within the buffer zone represented by the WMA where *An. quadriannulatus* larvae were found also had detectable numbers of impala, warthog, bushpig or a combination of two of these species. It therefore seems likely that local population viability, for *An. quadriannulatus* at least, is determined primarily by local conditions, particularly the availability of preferred hosts at quite fine spatial scales.

Second, small residual adult populations of the highly anthropophagic sibling species *An. gambiae* within the study area have been mapped out to remarkably small foci in and adjacent to the ILUMA WMA where unusual livelihood-related behaviours leave many people beyond the practical reach of LLIN use, allowing this vector safe access to human blood [82]. In stark contrast to the ubiquitous distribution of *An. arabiensis*, not a single adult specimen of *An. gambiae* was caught in NNP [3] illustrating how a human-specialized mosquito unable to exploit wild hosts was tightly restricted to settled areas and could not disperse beyond them in detectable numbers.

Third, each individual *An. arabiensis* larva collected at camp 40, located 47 km inside NNP from the park boundary (Fig 1) was found in a separate aquatic habitat, strongly suggesting that they originated from more than one mother. Furthermore, another 61 *An. arabiensis* larvae were collected in an impromptu purposive survey at *Mseguni* at the end of the study (Fig 1), which is located even further away from humans and cattle within NNP than camp 40. This strongly suggests the existence of a well-established population that acquire bloodmeals locally, rather than an otherwise non-viable *sink* population sustained by external *source* populations [78,79] outside the park.

### Implications of wild *Anopheles arabiensis* refuge populations living inside conservation areas

It was clear that unauthorised human and livestock encroachment was extensive throughout most of the ILUMA WMA and had a substantial negative impact on the availability and distribution of wild animals [67,70]. In particular, illegal cattle herding represents an influx of preferred blood sources for *An. arabiensis* and all major forms of encroachment reduce the availability of wild mammalian blood sources, thus displacing the non-vector *An. quadriannulatus* in favour of the malaria vector it competes with. If improved management of the WMA can be achieved in the future, to effectively control unauthorised human activities and facilitate the return of wild animals, a natural reduction of *An. arabiensis* in favour of *An. quadriannulatus* might well follow. Therefore, natural competitive suppression of this important malaria vector might represent a previously undocumented form of natural capital that arises from supporting and protecting conservation areas.

However, while it may be possible for *An. quadriannulatus* to competitively suppress *An. arabiensis* in conservation areas to some extent, the latter vector species was clearly not completely displaced from fully intact natural habitats lacking cattle or humans, even in the wildest areas with plenty of the wild herbivores the former species appears to prefer. This highlights how the behavioural plasticity of *An. arabiensis* allows it to extend its ecological niche deep inside wild areas, where they can even compete for resources against this much more zoophagic member of the same complex. Furthermore, the apparent existence of such refugia populations of *An. arabiensis* in these remote, well protected ecosystems confirms the initial hypothesis that alternative blood resources create an additional population-stabilising portfolio effect [28] for this species, further emphasizing the major vector control challenges posed by its notoriously flexible feeding behaviours [7,11,13,22].

Given that *An. arabiensis* were clearly and robustly the dominant competitive species wherever any humans or cattle whatsoever were found across the study area, and a refuge population of this species even exists deep inside NNP where there are none, the prospects for eliminating this key vector of residual malaria transmission with conventional human-centred vector control measures appear remote. Not only are wild refugia populations feeding upon wild animals largely out of the reach of current first-choice LLIN and IRS interventions that protect humans where and when they sleep indoors, they may also limit the population suppression effects [7] of new complementary approaches like spatial repellents and veterinary endectocides [127] that respectively extend insecticidal coverage to humans and livestock outdoors [128]. Furthermore, in parts of neighbouring Zambia where sympatric populations of *An. quadriannulatus* and *An. arabiensis* occur in domesticated settings, it seems that the former is much more vulnerable to insecticide attack than the latter, as evidenced by an abrupt species composition shift towards the latter vector species immediately after effective scale up of IRS [129]. It might therefore be difficult to target adult *An. arabiensis* with insecticide-based interventions without also affecting *An. quadriannulatus* populations at least as much.

On the other hand, two notable historical examples of successful elimination of this species from Brazil [111,130,131] (Retrospectively confirmed as *An. arabiensis* by molecular techniques [132]), and Egypt [133] (By far the most likely culprit based on ecological niche mapping [3,98]), were primarily accomplished through comprehensive larvicide application. While this might appear to suggest *An. arabiensis* may be tackled decisively at source as larvae, before they can express the evasive behaviours that make the adults so difficult to control, some important caveats merit interpretation. In both these examples of successful elimination, *An. arabiensis* was an invasive species that had established itself within a limited, albeit quite extensive new geographic range, within which it proved possible to contain, suppress and eventually eliminate. It is very difficult to envisage such “scorched earth” larval source management campaigns being feasible or affordable across the vast natural range of this species in Africa, especially if that includes refuge populations in the many large tracts of largely uninhabited wilderness scattered across the continent.

On a more positive note, although wild refugia populations deep inside NNP pose clear challenges for vector elimination, they also provide a potential opportunity for reversing the deleterious effects of pyrethroid resistance on the progress of malaria control to date [32–40]. Self-sustaining populations that do not come into contact with humans, livestock or agriculture, and are therefore free from the selective pressures of pesticides, may represent invaluable reservoirs of relatively unmodified mosquito genomes, complete with original wild-type insecticide susceptibility traits that would otherwise have been lost from the natural gene pool. Such refugia from selective pressure are considered crucial to successful insecticide resistance management over the long term, with theory increasingly backed up by experience of large-scale practice [29–31]. The existence of such large reservoirs of wild-type genomes that have never been bottlenecked by insecticide pressure, might even enable insecticide resistance management strategies that exploit astute insecticide combinations to select back these traits [134] in neighbouring populations living alongside people, thereby restoring the effectiveness of current [135] and future [136–140] interventions that remain dependent on this exceptionally useful insecticide class. It is therefore important that future studies of such refuge vector populations inside conserved wilderness areas also characterize their genomic diversity and resistance traits.

## Conclusions

While only *An. arabiensis* was found in fully domesticated ecosystems, its non-vector sibling species *An. quadriannulatus* occurred in conserved areas and dominated the best conserved natural ecosystems. The importance of humans and cattle to *An. arabiensis* as its two known preferred hosts [18,19] was confirmed by its clear positive association with signs of local activity by these two mammalian host species. The relative abundance of *An. quadriannulatus* increased with distance inside NNP and away from human settlements and was positively associated with activities of impala, warthog and perhaps bushpig, specifically implicating all three as possible preferred blood hosts. While abundant impala and lack of humans or cattle in fully intact acacia savannah within NNP apparently allowed it to dominate *An. arabiensis*, presence of warthog at two locations inside ILUMA seemed to provide it with a foothold in miombo woodlands well inside the WMA, despite considerable encroachment there by people and livestock. While this antelope is unrelated to the two implicated suids, all three are non-migratory residents of small home ranges with perennial surface water, representing likely preferred hosts for *An. quadriannulatus* that are widespread across extensive natural ecosystems all year round. Despite dominance of *An. quadriannulatus* in well-conserved areas, *An. arabiensis* was even found in absolutely intact natural environments >40km inside NNP, suggesting it can survive on blood from one or more unidentified wild species. Self-sustaining refuge populations of *An. arabiensis* inside conservation areas, supported by wild blood hosts that are fundamentally beyond the reach of insecticidal interventions targeted at humans or their livestock, may confound efforts to eliminate this key malaria vector but also enhance the success of insecticide resistance management strategies over the long term. This new approach to indirectly identifying their most commonly utilized blood sources may also be particularly applicable to an unprecedented diversity of zoophagic mosquitoes, enabling the incrimination of possible bridge vector species potentially capable of mediating pathogen spillover from wildlife reservoirs into livestock and/or human populations.

## Supporting information

S1 Appendix Detailed explanation of study area, camp location selection and field logistics. The rationale for selecting camp locations which acted as the sampling frame for the study (Fig 1), and the logistics and day-to-day procedures of visiting these camps. Includes a table of camp locations, and photographs illustrating some camps as examples.
(PDF)

S2 Appendix Detailed larval survey protocol. (PDF)

S3 Appendix Validation of field identification methodology used for larvae from the *Anopheles gambiae* complex using a general linear mixed model. (PDF)

S4 Appendix Assessing natural ecosystem integrity: Methodological approach and validation of a subjective natural ecosystem integrity index (SNEII). (PDF)

S5 Data All the data from the larval habitat occupancy and habitat attribute surveys. (CSV)

S6 Appendix Procedures for data entry, cleaning, preparation and detailed data analysis. (PDF)

S7 Data The species composition data from the larvae that were collected in the field, and all the data from the complimentary humans, livestock and wildlife surveys. (CSV)

S8 Appendix Conceptual framework for regression analysis of the association between *Anopheles gambiae* sibling species composition and indicators of activity by diverse mammalian species that could act as potential sources of blood for mosquitoes. (PDF)

S9 Fig The detection frequency of humans plotted against the detection frequency of cattle herds at each camp number, demonstrating a strong linear correlation, as tested using a Pearson’s linear correlation test. (PDF)

S10 Appendix Habitat characteristics and occupancy by mosquito larvae, particularly those from the *Anopheles gambiae* species complex. (PDF)

S11 Text Defining competition, competitive displacement and competitive co-existence in strict ecological terms. (PDF)

S12 Fig Preliminary *Anopheles gambiae* complex species composition results from F0 adults raised from field collected larvae. (PDF)

## References

[1] Coetzee M, Craig M, le Sueur D. Distribution of African malaria mosquitoes belonging to the *Anopheles gambiae* complex. Parasitol Today. 2000;16(2):74–7. doi: 10.1016/s0169-4758(99)01563-x 10652493

[2] Levine RS, Peterson AT, Benedict MQ. Geographic and ecologic distributions of the *Anopheles gambiae* complex predicted using a genetic algorithm. Am J Trop Med Hyg. 2004;70(2):105–9. doi: 10.4269/ajtmh.2004.70.105 14993618

[3] Moffett A, Shackelford N, Sarkar S. Malaria in Africa: vector species’ niche models and relative risk maps. PLoS One. 2007;2(9):e824. doi: 10.1371/journal.pone.0000824 17786196 PMC1950570

[4] White GB. *Anopheles gambiae* complex and disease transmission in Africa. Trans R Soc Trop Med Hyg. 1974;68(4):278–301. doi: 10.1016/0035-9203(74)90035-2 4420769

[5] Kiszewski A, Mellinger A, Spielman A, Malaney P, Sachs SE, Sachs J. A global index representing the stability of malaria transmission. Am J Trop Med Hyg. 2004;70(5):486–98. doi: 10.4269/ajtmh.2004.70.486 15155980

[6] Sinka ME, Bangs MJ, Manguin S, Rubio-Palis Y, Chareonviriyaphap T, Coetzee M, et al. A global map of dominant malaria vectors. Parasit Vectors. 2012;5:69. doi: 10.1186/1756-3305-5-69 22475528 PMC3349467

[7] Killeen GF, Kiware SS, Okumu FO, Sinka ME, Moyes CL, Massey NC, et al. Going beyond personal protection against mosquito bites to eliminate malaria transmission: population suppression of malaria vectors that exploit both human and animal blood. BMJ Glob Health. 2017;2(2):e000198. doi: 10.1136/bmjgh-2016-000198 28589015 PMC5444054

[8] Russell TL, Lwetoijera DW, Maliti D, Chipwaza B, Kihonda J, Charlwood JD, et al. Impact of promoting longer-lasting insecticide treatment of bed nets upon malaria transmission in a rural Tanzanian setting with pre-existing high coverage of untreated nets. Malar J. 2010;9:187. doi: 10.1186/1475-2875-9-187 20579399 PMC2902500

[9] Bayoh MN, Mathias DK, Odiere MR, Mutuku FM, Kamau L, Gimnig JE, et al. *Anopheles gambiae*: historical population decline associated with regional distribution of insecticide-treated bed nets in western Nyanza Province, Kenya. Malar J. 2010;9:62. doi: 10.1186/1475-2875-9-62 20187956 PMC2838909

[10] Killeen GF, Seyoum A, Sikaala C, Zomboko AS, Gimnig JE, Govella NJ, et al. Eliminating malaria vectors. Parasit Vectors. 2013;6:172. doi: 10.1186/1756-3305-6-172 23758937 PMC3685528

[11] Killeen GF, Govella NJ, Lwetoijera DW, Okumu FO. Most outdoor malaria transmission by behaviourally-resistant *Anopheles arabiensis* is mediated by mosquitoes that have previously been inside houses. Malar J. 2016;15:225. doi: 10.1186/s12936-016-1280-z 27093890 PMC4837512

[12] Durnez L, Coosemans M. Residual transmission of malaria: an old issue for new approaches. In: Manguin S, editor. Anopheles mosquitoes: new insights into malaria vectors. Rijeka: InTech; 2013.

[13] Killeen GF. Characterizing, controlling and eliminating residual malaria transmission. Malar J. 2014;13:1–22. doi: 10.1186/1475-2875-13-330 25149656 PMC4159526

[14] WHO. Malaria terminology, 2021 update. Geneva: World Health Organization; 2021.

[15] WHO. Control of residual malaria parasite transmission: guidance note. Geneva: World Health Organization; 2014.

[16] Gillies MT, Coetzee M. A supplement to the Anophelinae of Africa South of the Sahara. South African Institute for Medical Research; 1987.

[17] Gillies MT, De Meillon B. The Anophelinae of Africa South of the Sahara (Ethiopian Zoogeographical Region). 2nd ed. South African Institute for Medical Research; 1968.

[18] White G, Magayuka SA, Boreham P. Comparative studies on sibling species of the *Anopheles gambiae* Giles complex (Dipt., Culicidae): bionomics and vectorial activity of species A and species B at Segera, Tanzania. Bull Entomol Res. 1972;62(2):295–317. doi: 10.1017/S0007485300047738

[19] Killeen GF, McKenzie FE, Foy BD, Bøgh C, Beier JC. The availability of potential hosts as a determinant of feeding behaviours and malaria transmission by African mosquito populations. Trans R Soc Trop Med Hyg. 2001;95(5):469–76. doi: 10.1016/s0035-9203(01)90005-7 11706651 PMC2483839

[20] Govella NJ, Chaki PP, Killeen GF. Entomological surveillance of behavioural resilience and resistance in residual malaria vector populations. Malar J. 2013;12:124. doi: 10.1186/1475-2875-12-124 23577656 PMC3637503

[21] Elliott R. The influence of vector behavior on malaria transmission. Am J Trop Med Hyg. 1972;21(5):755–63. doi: 10.4269/ajtmh.1972.21.755 4561523

[22] Killeen GF, Chitnis N. Potential causes and consequences of behavioural resilience and resistance in malaria vector populations: a mathematical modelling analysis. Malar J. 2014;13:97. doi: 10.1186/1475-2875-13-97 24629066 PMC3995604

[23] Ayala FJ, Coluzzi M. Chromosome speciation: humans, *Drosophila*, and mosquitoes. Proc Natl Acad Sci. 2005;102(suppl_1):6535–42. doi: 10.1073/pnas.0501847102 15851677 PMC1131864

[24] Prior A, Torr SJ. Host selection by *Anopheles arabiensis* and *An. quadriannulatus* feeding on cattle in Zimbabwe. Med Vet Entomol. 2002;16(2):207–13. doi: 10.1046/j.1365-2915.2002.00367.x 12109716

[25] Braack LE, Coetzee M, Hunt RH, Biggs H, Cornel A, Gericke A. Biting pattern and host-seeking behavior of *Anopheles arabiensis* (Diptera: Culicidae) in northeastern South Africa. J Med Entomol. 1994;31(3):333–9. doi: 10.1093/jmedent/31.3.333 8057306

[26] Munhenga G, Brooke BD, Chirwa TF, Hunt RH, Coetzee M, Govender D, et al. Evaluating the potential of the sterile insect technique for malaria control: relative fitness and mating compatibility between laboratory colonized and a wild population of *Anopheles arabiensis* from the Kruger National Park, South Africa. Parasit Vectors. 2011;4:208. doi: 10.1186/1756-3305-4-208 22041133 PMC3216276

[27] Munhenga G, Brooke BD, Spillings B, Essop L, Hunt RH, Midzi S, et al. Field study site selection, species abundance and monthly distribution of anopheline mosquitoes in the northern Kruger National Park, South Africa. Malar J. 2014;13:27. doi: 10.1186/1475-2875-13-27 24460920 PMC3925985

[28] Killeen GF, Reed TE. The portfolio effect cushions mosquito populations and malaria transmission against vector control interventions. Malar J. 2018;17(1):291. doi: 10.1186/s12936-018-2441-z 30097031 PMC6086012

[29] Mangan R, Bussière LF, Polanczyk RA, Tinsley MC. Increasing ecological heterogeneity can constrain biopesticide resistance evolution. Trends Ecol Evol. 2023;38(7):605–14. doi: 10.1016/j.tree.2023.01.012 36906434

[30] Hobbs NP, Weetman D, Hastings IM. Insecticide resistance management strategies for public health control of mosquitoes exhibiting polygenic resistance: A comparison of sequences, rotations, and mixtures. Evol. Appl. 2023; 16(4):936–59.doi: 10.1111/eva.13546 37124088 PMC10130562

[31] Tabashnik BE, Fabrick JA, Carrière Y. Global Patterns of Insect Resistance to Transgenic Bt Crops: The First 25 Years. J Econ Entomol. 2023;116(2):297–309. doi: 10.1093/jee/toac183 36610076

[32] Ranson H, Lissenden N. Insecticide resistance in african anopheles mosquitoes: a worsening situation that needs urgent action to maintain malaria control. Trends Parasitol. 2016;32(3):187–96. doi: 10.1016/j.pt.2015.11.010 26826784

[33] Hemingway J, Ranson H, Magill A, Kolaczinski J, Fornadel C, Gimnig J, et al. Averting a malaria disaster: will insecticide resistance derail malaria control? Lancet. 2016;387(10029):1785–8. doi: 10.1016/S0140-6736(15)00417-1 26880124 PMC6215693

[34] Churcher TS, Lissenden N, Griffin JT, Worrall E, Ranson H. The impact of pyrethroid resistance on the efficacy and effectiveness of bednets for malaria control in Africa. Elife. 2016. doi: 10.7554/elife.16090 PMC5025277 27547988

[35] Hancock PA, Hendriks CJM, Tangena J-A, Gibson H, Hemingway J, Coleman M, et al. Mapping trends in insecticide resistance phenotypes in African malaria vectors. PLoS Biol. 2020;18(6):e3000633. doi: 10.1371/journal.pbio.3000633 32584814 PMC7316233

[36] Moyes CL, Athinya DK, Seethaler T, Battle KE, Sinka M, Hadi MP, et al. Evaluating insecticide resistance across African districts to aid malaria control decisions. Proc Natl Acad Sci U S A. 2020;117(36):22042–50. doi: 10.1073/pnas.2006781117 32843339 PMC7486715

[37] Mosha JF, Kulkarni MA, Lukole E, Matowo NS, Pitt C, Messenger LA, et al. Effectiveness and cost-effectiveness against malaria of three types of dual-active-ingredient long-lasting insecticidal nets (LLINs) compared with pyrethroid-only LLINs in Tanzania: a four-arm, cluster-randomised trial. Lancet. 2022;399(10331):1227–41. doi: 10.1016/S0140-6736(21)02499-5 35339225 PMC8971961

[38] Accrombessi M, Cook J, Dangbenon E, Yovogan B, Akpovi H, Sovi A, et al. Efficacy of pyriproxyfen-pyrethroid long-lasting insecticidal nets (LLINs) and chlorfenapyr-pyrethroid LLINs compared with pyrethroid-only LLINs for malaria control in Benin: a cluster-randomised, superiority trial. Lancet. 2023;401(10375):435–46. doi: 10.1016/S0140-6736(22)02319-4 36706778

[39] WHO. World malaria report. Geneva: World Health Organisation; 2023.

[40] Killeen GF, Sougoufara S. Getting ahead of insecticide-resistant malaria vector mosquitoes. Lancet. 2023;401(10375):410–1. doi: 10.1016/S0140-6736(23)00102-2 36706777

[41] Costanza R, d’Arge R, De Groot R, Farber S, Grasso M, Hannon B, et al. The value of the world’s ecosystem services and natural capital. Nature. 1997; 387(6630):253–60. doi: 10.1038/387253a0

[42] Ekins P, Simon S, Deutsch L, Folke C, De Groot R. A framework for the practical application of the concepts of critical natural capital and strong sustainability. Ecol Econ. 2003;44(2–3):165–85. doi: 10.1016/S0921-8009(02)00272-0

[43] Costanza R. Valuing natural capital and ecosystem services toward the goals of efficiency, fairness, and sustainability. Ecosyst Serv. 2020;43:101096. doi: 10.1016/j.ecoser.2020.101096

[44] Killeen GF. Control of malaria vectors and management of insecticide resistance through universal coverage with next-generation insecticide-treated nets. Lancet. 2020;395(10233):1394–400. doi: 10.1016/S0140-6736(20)30745-5 32304648

[45] Yang Y, Dong B, Xu H, Zheng X, Tian J, Heong K, et al. Decrease of insecticide resistance over generations without exposure to insecticides in Nilaparvata lugens (Hemipteran: Delphacidae). J Econ Entomol. 2014;107(4):1618–25. doi: 10.1603/ec13550 25195455

[46] Nkahe DL, Sonhafouo-Chiana N, Ndjeunia Mbiakop P, Kekeunou S, Mimpfoundi R, Awono-Ambene P, et al. Can the use of larviciding with biological compounds contribute in increasing Anopheles gambiae s.l. susceptibility to pyrethroid in a population expressing high resistance intensity?. Pestic Biochem Physiol. 2023;195:105569. doi: 10.1016/j.pestbp.2023.105569 37666599

[47] Penilla-Navarro P, Solis-Santoyo F, Lopez-Solis A, Rodriguez AD, Vera-Maloof F, Lozano S, et al. Pyrethroid susceptibility reversal in *Aedes aegypti*: A longitudinal study in Tapachula, Mexico. PLoS Negl Trop Dis. 2024;18(1):e0011369. doi: 10.1371/journal.pntd.0011369 38166129 PMC10786364

[48] Silva-Filha MH, Regis L. Reversal of low-level resistance to *Bacillus sphaericus* in a field population of the southern house mosquito (Diptera:Culicidae) from an urban area of Recife, Brazil. J Econ Entomol. 1997;90(2):299–303. doi: 10.1093/jee/90.2.299 9145030

[49] Bang Y, Jatanasen S, Tonn RJ. Development and reversion of DDT resistance in an Aedes aegypti population in Bangkok, Thailand. Bulletin of the World Health Organization. 1971;45(3):404. 5316918 PMC2427919

[50] Coluzzi M, Sabatini A, Petrarca V, Di Deco MA. Chromosomal differentiation and adaptation to human environments in the *Anopheles gambiae* complex. Trans R Soc Trop Med Hyg. 1979;73(5):483–97. doi: 10.1016/0035-9203(79)90036-1 394408

[51] Coluzzi M. Heterogeneities of the malaria vectorial system in tropical Africa and their significance in malaria epidemiology and control. Bull World Health Organ. 1984;62 Suppl(Suppl):107–13. 6335681 PMC2536202

[52] Tonnang HEZ, Kangalawe RYM, Yanda PZ. Predicting and mapping malaria under climate change scenarios: the potential redistribution of malaria vectors in Africa. Malar J. 2010;9:111. doi: 10.1186/1475-2875-9-111 20416059 PMC2873524

[53] Freyvogel TA, Kihaule PM. Report on a limited anopheline survey at Ifakara, South-Eastern Tanzania. Acta Trop. 1968;25(1):17–28. 4385521

[54] Smith T, Charlwood JD, Kihonda J, Mwankusye S, Billingsley P, Meuwissen J, et al. Absence of seasonal variation in malaria parasitaemia in an area of intense seasonal transmission. Acta Trop. 1993;54(1):55–72. doi: 10.1016/0001-706x(93)90068-m 8103627

[55] Smith T, Charlwood JD, Takken W, Tanner M, Spiegelhalter DJ. Mapping the densities of malaria vectors within a single village. Acta Trop. 1995;59(1):1–18. doi: 10.1016/0001-706x(94)00082-c 7785522

[56] Charlwood J, Smith T, Kihonda J, Heiz B, Billingsley P, Takken W. Density independent feeding success of malaria vectors (Diptera: Culicidae) in Tanzania. Bull Entom Res. 1995;85(1):29–35. doi: 10.1017/S0007485300051981

[57] Charlwood J, Kihonda J, Sama S, Billingsley P, Hadji H, Verhave J. The rise and fall of Anopheles arabiensis (Diptera: Culicidae) in a Tanzanian village. Bull Entom Res. 1995;85(1):37–44. doi: 10.1017/S0007485300051993

[58] Takken W, Charlwood J, Billingsley P, Gort G. Dispersal and survival of *Anopheles funestus* and *A. gambiae* s.l. (Diptera: Culicidae) during the rainy season in southeast Tanzania. Bull Entom Res. 1998;88(5):561–6. doi: 10.1017/S0007485300026080

[59] Charlwood J. Vectorial capacity, species diversity and population cycles of anopheline mosquitoes (Diptera: Culicidae) from indoor light-trap collections in a house in southeastern Tanzania. Afr Entomol. 1997;5(1):93–101.

[60] Charlwood JD, Vij R, Billingsley PF. Dry season refugia of malaria-transmitting mosquitoes in a dry savannah zone of east Africa. Am J Trop Med Hyg. 2000;62(6):726–32. doi: 10.4269/ajtmh.2000.62.726 11304064

[61] Killeen GF, Kihonda J, Lyimo E, Oketch FR, Kotas ME, Mathenge E, et al. Quantifying behavioural interactions between humans and mosquitoes: evaluating the protective efficacy of insecticidal nets against malaria transmission in rural Tanzania. BMC Infect Dis. 2006;6:161. doi: 10.1186/1471-2334-6-161 17096840 PMC1657018

[62] Killeen GF, Tami A, Kihonda J, Okumu FO, Kotas ME, Grundmann H, et al. Cost-sharing strategies combining targeted public subsidies with private-sector delivery achieve high bednet coverage and reduced malaria transmission in Kilombero Valley, southern Tanzania. BMC Infect Dis. 2007;7:121. doi: 10.1186/1471-2334-7-121 17961211 PMC2211306

[63] Drakeley CJ, Akim NI, Sauerwein RW, Greenwood BM, Targett GA. Estimates of the infectious reservoir of *Plasmodium falciparum* malaria in The Gambia and in Tanzania. Trans R Soc Trop Med Hyg. 2000;94(5):472–6. doi: 10.1016/s0035-9203(00)90056-7 11132369

[64] Russell TL, Govella NJ, Azizi S, Drakeley CJ, Kachur SP, Killeen GF. Increased proportions of outdoor feeding among residual malaria vector populations following increased use of insecticide-treated nets in rural Tanzania. Malar J. 2011;10:80. doi: 10.1186/1475-2875-10-80 21477321 PMC3084176

[65] Lwetoijera DW, Harris C, Kiware SS, Dongus S, Devine GJ, McCall PJ, et al. Increasing role of Anopheles funestus and *Anopheles arabiensis* in malaria transmission in the Kilombero Valley, Tanzania. Malar J. 2014;13:331. doi: 10.1186/1475-2875-13-331 25150840 PMC4150941

[66] Duggan LM, Tarimo LJ, Walsh KA, Kavishe DR, Crego R, Manase E. Afr J Ecol. 2024;62(3):e13309. doi: 10.1111/aje.13309

[67] Duggan LM, Walsh KA, Tarimo LJ, Kavishe DR, Crego RD, Elisa M, et al. A subjective and intuitive approach to rapid, holistic assessment of natural ecosystem integrity across a community-managed conservation area in Southern Tanzania. Ecol Evol. 2025;15(3):e70872. doi: 10.1002/ece3.70872 40034427 PMC11872596

[68] WalshKA. Blood host preferences and competitive inter-species dynamics within an African malaria vector species complex inferred from signs of animal activity around aquatic larval habitats distributed across a gradient of fully domesticated to fully pristine ecosystems in southern Tanzania. University College Cork; 2023. https://hdl.handle.net/10468/1592610.1371/journal.pone.034467041894457

[69] Kavishe DR, Msoffe RV, Malika GZ, Walsh KA, Duggan LM, Tarimo LJ, et al. A self‐cooling self‐humidifying mosquito carrier backpack for transporting live adult mosquitoes on foot over long distances under challenging field conditions. Medical and Veterinary Entomology. 2025;39(1):171–86. doi: 10.1111/mve.12771

[70] Duggan LM. The influence of community-defined land use plans and de facto land use practices on the relative abundance and distribution of large wild mammals in a community-based wildlife management area in southern Tanzania. University College Cork; 2023. https://hdl.handle.net/10468/15928

[71] Service MW. Mosquito (Diptera: Culicidae) dispersal--the long and short of it. J Med Entomol. 1997;34(6):579–88. doi: 10.1093/jmedent/34.6.579 9439109

[72] Kaufmann C, Briegel H. Flight performance of the malaria vectors *Anopheles gambiae* and *Anopheles atroparvus*. J Vector Ecol. 2004;29(1):140–53. 15266751

[73] Kiware SS, Russell TL, Mtema ZJ, Malishee AD, Chaki P, Lwetoijera D, et al. A generic schema and data collection forms applicable to diverse entomological studies of mosquitoes. Source Code Biol Med. 2016;11:4. doi: 10.1186/s13029-016-0050-1 27022408 PMC4809029

[74] Holstein MH. Biology of Anopheles gambiae: research in French west Africa. World Health Organization; 1954.

[75] Koenraadt CJM, Takken W. Cannibalism and predation among larvae of the *Anopheles gambiae* complex. Med Vet Entomol. 2003;17(1):61–6. doi: 10.1046/j.1365-2915.2003.00409.x 12680927

[76] Koenraadt C, Majambere S, Hemerik L, Takken W. The effects of food and space on the occurrence of cannibalism and predation among larvae of *Anopheles gambiae* s.l. Entomol Exp Appl. 2004;112(2):125–34. doi: 10.1111/j.0013-8703.2004.00186.x

[77] Scott JA, Brogdon WG, Collins FH. Identification of single specimens of the *Anopheles gambiae* complex by the polymerase chain reaction. Am J Trop Med Hyg. 1993;49(4):520–9. doi: 10.4269/ajtmh.1993.49.520 8214283

[78] Pulliam HR. Sources, sinks, and population regulation. Am Nat. 1988;132(5):652–61.

[79] Dias PC. Sources and sinks in population biology. Trends Ecol Evol. 1996;11(8):326–30. doi: 10.1016/0169-5347(96)10037-9 21237863

[80] Charlwood JD, Edoh D. Polymerase chain reaction used to describe larval habitat use by *Anopheles gambiae* complex (Diptera: Culicidae) in the environs of Ifakara, Tanzania. J Med Entomol. 1996;33(2):202–4. doi: 10.1093/jmedent/33.2.202 8742521

[81] Minakawa N, Mutero CM, Githure JI, Beier JC, Yan G. Spatial distribution and habitat characterization of anopheline mosquito larvae in Western Kenya. Am J Trop Med Hyg. 1999;61(6):1010–6. doi: 10.4269/ajtmh.1999.61.1010 10674687

[82] Kavishe DR, Tarimo LJ, Msoffe RV, Walsh KA, Duggan LM, et al. Persistence of residual *Anopheles gambiae* populations associated with frontier human settlements and nocturnal livelihood-related activities at the fringes of a large conservation area in southern Tanzania. bioRxiv. 2025. doi: 10.1101/2025.10.24.684313

[83] Estes RD. The behavior guide to African mammals: including hoofed mammals, carnivores, primates. University of California Press; 2012.

[84] Maia MF, Kreppel K, Mbeyela E, Roman D, Mayagaya V, Lobo NF, et al. A crossover study to evaluate the diversion of malaria vectors in a community with incomplete coverage of spatial repellents in the Kilombero Valley, Tanzania. Parasit Vectors. 2016;9:451. doi: 10.1186/s13071-016-1738-4 27527601 PMC4986272

[85] Kavishe DR, Walsh KA, Msoffe RV, Duggan LM, Tarimo LJ, et al. Med Vet Entomol. 2025;39(4):726–40. doi: 10.1111/mve.12813

[86] Diabaté A, Dabire RK, Kim EH, Dalton R, Millogo N, Baldet T, et al. Larval development of the molecular forms of *Anopheles gambiae* (Diptera: Culicidae) in different habitats: a transplantation experiment. J Med Entomol. 2005;42(4):548–53. doi: 10.1093/jmedent/42.4.548 16119542

[87] Simard F, Ayala D, Kamdem GC, Pombi M, Etouna J, Ose K, et al. Ecological niche partitioning between Anopheles gambiae molecular forms in Cameroon: the ecological side of speciation. BMC Ecol. 2009;9:17. doi: 10.1186/1472-6785-9-17 19460146 PMC2698860

[88] Gimnig JE, Ombok M, Kamau L, Hawley WA. Characteristics of larval anopheline (Diptera: Culicidae) habitats in Western Kenya. J Med Entomol. 2001;38(2):282–8. doi: 10.1603/0022-2585-38.2.282 11296836

[89] Edillo FE, Touré YT, Lanzaro GC, Dolo G, Taylor CE. Spatial and habitat distribution of *Anopheles gambiae* and *Anopheles arabiensis* (Diptera: Culicidae) in Banambani village, Mali. J Med Entomol. 2002;39(1):70–7. doi: 10.1603/0022-2585-39.1.70 11931274

[90] Gillies MT, Smith A. The effect of a residual house-spraying campaign in East Africa on species balance in the *Anopheles funestus* group. The replacement of *A. funestus* Giles by *A. rivulorum* Leeson. Bull Entomol Res. 1960;51(2):243–52. doi: 10.1017/S0007485300057953

[91] Ho BC, Ewert A, Chew LM. Interspecific competition among *Aedes aegypti*, *Ae. albopictus*, and *Ae. triseriatus* (Diptera: Culicidae): larval development in mixed cultures. J Med Entomol. 1989;26(6):615–23. doi: 10.1093/jmedent/26.6.615 2585456

[92] Juliano SA. Species introduction and replacement among mosquitoes: interspecific resource competition or apparent competition? Ecology. 1998; 79(1):255–68. doi: 10.1890/0012-9658(1998)079[0255:SIARAM]2.0.CO;2

[93] Gimnig JE, Ombok M, Otieno S, Kaufman MG, Vulule JM, Walker ED. Density-dependent development of *Anopheles gambiae* (Diptera: Culicidae) larvae in artificial habitats. J Med Entomol. 2002;39(1):162–72. doi: 10.1603/0022-2585-39.1.162 11931252

[94] Muriu SM, Coulson T, Mbogo CM, Godfray HCJ. Larval density dependence in *Anopheles gambiae* s.s., the major African vector of malaria. J Anim Ecol. 2013;82(1):166–74. doi: 10.1111/1365-2656.12002 23163565 PMC5373432

[95] Barreaux AMG, Stone CM, Barreaux P, Koella JC. The relationship between size and longevity of the malaria vector *Anopheles gambiae* (s.s.) depends on the larval environment. Parasit Vectors. 2018;11(1):485. doi: 10.1186/s13071-018-3058-3 30157916 PMC6114828

[96] Smith A. Malaria in the Taveta area of Kenya and Tanganyika. Part III. Entomological findings three years after the spraying period. East Afr Med J. 1962;39(9):553–64. 13993113

[97] Mutuku FM, King CH, Mungai P, Mbogo C, Mwangangi J, Muchiri EM, et al. Impact of insecticide-treated bed nets on malaria transmission indices on the south coast of Kenya. Malar J. 2011;10:356. doi: 10.1186/1475-2875-10-356 22165904 PMC3322380

[98] Sinka ME, Golding N, Massey NC, Wiebe A, Huang Z, Hay SI, et al. Modelling the relative abundance of the primary African vectors of malaria before and after the implementation of indoor, insecticide-based vector control. Malar J. 2016;15:142. doi: 10.1186/s12936-016-1187-8 26945997 PMC4779559

[99] Derua YA, Alifrangis M, Hosea KM, Meyrowitsch DW, Magesa SM, Pedersen EM, et al. Change in composition of the *Anopheles gambiae* complex and its possible implications for the transmission of malaria and lymphatic filariasis in north-eastern Tanzania. Malar J. 2012;11:188. doi: 10.1186/1475-2875-11-188 22681999 PMC3469399

[100] Mwalimu CD, Kiware S, Nshama R, Derua Y, Machafuko P, Gitanya P, et al. Dynamics of malaria vector composition and *Plasmodium falciparum* infection in mainland Tanzania: 2017-2021 data from the national malaria vector entomological surveillance. Malar J. 2024;23(1):29. doi: 10.1186/s12936-024-04849-7 38243220 PMC10797900

[101] Msugupakulya BJ, Urio NH, Jumanne M, Ngowo HS, Selvaraj P, Okumu FO, et al. Changes in contributions of different *Anopheles* vector species to malaria transmission in east and southern Africa from 2000 to 2022. Parasit Vectors. 2023;16(1):408. doi: 10.1186/s13071-023-06019-1 37936155 PMC10631025

[102] Lounibos LP. Competitive displacement and reduction. J Am Mosq Control Assoc. 2007;23(2 Suppl):276–82. doi: 10.2987/8756-971X(2007)23[276:CDAR]2.0.CO;2 17853612 PMC2212597

[103] Pates HV, Takken W, Curtis CF, Huisman PW, Akinpelu O, Gill GS. Unexpected anthropophagic behaviour in *Anopheles quadriannulatus*. Med Vet Entomol. 2001;15(3):293–8. doi: 10.1046/j.0269-283x.2001.00310.x 11583447

[104] Torr SJ, Della Torre A, Calzetta M, Costantini C, Vale GA. Towards a fuller understanding of mosquito behaviour: use of electrocuting grids to compare the odour-orientated responses of *Anopheles arabiensis* and *An. quadriannulatus* in the field. Med Vet Entomol. 2008;22(2):93–108. doi: 10.1111/j.1365-2915.2008.00723.x 18498608

[105] Seyoum A, Sikaala CH, Chanda J, Chinula D, Ntamatungiro AJ, Hawela M, et al. Human exposure to anopheline mosquitoes occurs primarily indoors, even for users of insecticide-treated nets in Luangwa Valley, South-east Zambia. Parasit Vectors. 2012;5:101. doi: 10.1186/1756-3305-5-101 22647493 PMC3432592

[106] Service M. A critical review of procedures for sampling populations of adult mosquitoes. Bull Entomol Res. 1977;67(3):343–82. doi: 10.1017/S0007485300011184

[107] Service M. Mosquito ecology: field sampling methods. 3rd ed. Springer; 1993.

[108] Burkot TR, Russell TL, Reimer LJ, Bugoro H, Beebe NW, Cooper RD, et al. Barrier screens: a method to sample blood-fed and host-seeking exophilic mosquitoes. Malar J. 2013;12:49. doi: 10.1186/1475-2875-12-49 23379959 PMC3574015

[109] Killeen GF, Chaki PP, Reed TE, Moyes CL, Govella NJ. Entomological surveillance as a cornerstone of malaria elimination: a critical appraisal. Towards malaria elimination-a leap forward. InTech. 2018.

[110] Yan J, Gangoso L, Ruiz S, Soriguer R, Figuerola J, Martínez-de la Puente J. Understanding host utilization by mosquitoes: determinants, challenges and future directions. Biol Rev Camb Philos Soc. 2021;96(4):1367–85. doi: 10.1111/brv.12706 33686781

[111] Soper FL, Wilson DB. Anopheles gambiae in Brazil, 1930 to 1940. Rockefeller Foundation; 1943.

[112] Port G, Boreham P, Bryan JH. The relationship of host size to feeding by mosquitoes of the *Anopheles gambiae* Giles complex (Diptera: Culicidae). Bull Entomol Res. 1980;70(1):133–44. doi: 10.1017/S0007485300009834

[113] Takken W, Eling W, Hooghof J, Dekker T, Hunt R, Coetzee M. Susceptibility of *Anopheles quadriannulatus* Theobald (Diptera: Culicidae) to *Plasmodium falciparum*. Trans R Soc Trop Med Hyg. 1999;93(6):578–80. doi: 10.1016/s0035-9203(99)90054-8 10717736

[114] Lehane MJ. The biology of blood-sucking in insects. 2nd ed. Cambridge University Press; 2005.

[115] Smith T, Maire N, Dietz K, Killeen GF, Vounatsou P, Molineaux L, et al. Relationship between the entomologic inoculation rate and the force of infection for *Plasmodium falciparum* malaria. Am J Trop Med Hyg. 2006;75(2 Suppl):11–8. doi: 10.4269/ajtmh.2006.75.2_suppl.0750011 16931811

[116] Takken W, Verhulst NO. Host preferences of blood-feeding mosquitoes. Annu Rev Entomol. 2013;58:433–53. doi: 10.1146/annurev-ento-120811-153618 23020619

[117] Lyimo IN, Ferguson HM. Ecological and evolutionary determinants of host species choice in mosquito vectors. Trends Parasitol. 2009;25(4):189–96. doi: 10.1016/j.pt.2009.01.005 19269900

[118] Silver JB. Mosquito Ecology: Field Sampling Methods. 3rd ed. Springer Science & Business Media; 2007.

[119] Soghigian J, Sither C, Justi SA, Morinaga G, Cassel BK, Vitek CJ, et al. Phylogenomics reveals the history of host use in mosquitoes. Nat Commun. 2023;14(1):6252. doi: 10.1038/s41467-023-41764-y 37803007 PMC10558525

[120] Janecek LL, Honeycutt RL, Adkins RM, Davis SK. Mitochondrial gene sequences and the molecular systematics of the artiodactyl subfamily bovinae. Mol Phylogenet Evol. 1996;6(1):107–19. doi: 10.1006/mpev.1996.0063 8812311

[121] Crabtree MB, Kading RC, Mutebi J-P, Lutwama JJ, Miller BR. Identification of host blood from engorged mosquitoes collected in western Uganda using cytochrome oxidase I gene sequences. J Wildl Dis. 2013;49(3):611–26. doi: 10.7589/2012-08-213 23778610

[122] Lindsay SW, Parson L, Thomas CJ. Mapping the ranges and relative abundance of the two principal African malaria vectors, *Anopheles gambiae sensu stricto* and *An. arabiensis*, using climate data. Proc Biol Sci. 1998;265(1399):847–54. doi: 10.1098/rspb.1998.0369 9633110 PMC1689061

[123] Gillies MT. Studies on the dispersion and survival of *Anopheles gambiae* Giles in East Africa, by means of marking and release experiments. Bull Entomol Res. 1961;52(1):99–127. doi: 10.1017/S0007485300055309

[124] Huestis DL, Dao A, Diallo M, Sanogo ZL, Samake D, Yaro AS, et al. Windborne long-distance migration of malaria mosquitoes in the Sahel. Nature. 2019;574(7778):404–8. doi: 10.1038/s41586-019-1622-4 31578527 PMC11095661

[125] Florio J, Verú LM, Dao A, Yaro AS, Diallo M, Sanogo ZL, et al. Diversity, dynamics, direction, and magnitude of high-altitude migrating insects in the Sahel. Sci Rep. 2020;10(1):20523. doi: 10.1038/s41598-020-77196-7 33239619 PMC7688652

[126] Atieli HE, Zhou G, Zhong D, Wang X, Lee M c, Yaro AS. Wind-assisted high-altitude dispersal of mosquitoes and other insects in East Africa. J Med Entomol. 2023;60(4):698–707. doi: 10.1093/jme/tjad033 37094808 PMC10337859

[127] Killeen GF, Tatarsky A, Diabate A, Chaccour CJ, Marshall JM, Okumu FO, et al. Developing an expanded vector control toolbox for malaria elimination. BMJ Glob Health. 2017;2(2):e000211. doi: 10.1136/bmjgh-2016-000211 28589022 PMC5444090

[128] Killeen GF, Marshall JM, Kiware SS, South AB, Tusting LS, Chaki PP, et al. Measuring, manipulating and exploiting behaviours of adult mosquitoes to optimise malaria vector control impact. BMJ Glob Health. 2017;2(2):e000212. doi: 10.1136/bmjgh-2016-000212 28589023 PMC5444085

[129] Chinula D, Hamainza B, Chizema E, Kavishe DR, Sikaala CH, Killeen GF. Proportional decline of *Anopheles quadriannulatus* and increased contribution of An. arabiensis to the *An. gambiae* complex following introduction of indoor residual spraying with pirimiphos-methyl: an observational, retrospective secondary analysis of pre-existing data from south-east Zambia. Parasit Vectors. 2018;11(1):544. doi: 10.1186/s13071-018-3121-0 30305147 PMC6180389

[130] Killeen GF, Fillinger U, Kiche I, Gouagna LC, Knols BGJ. Eradication of *Anopheles gambiae* from Brazil: lessons for malaria control in Africa? Lancet Infect Dis. 2002;2(10):618–27. doi: 10.1016/s1473-3099(02)00397-3 12383612

[131] Killeen GF. Following in Soper’s footsteps: northeast Brazil 63 years after eradication of *Anopheles gambiae*. Lancet Infect Dis. 2003;3(10):663–6. doi: 10.1016/s1473-3099(03)00776-x 14522266

[132] Parmakelis A, Russello MA, Caccone A, Marcondes CB, Costa J, Forattini OP, et al. Historical analysis of a near disaster: *Anopheles gambiae* in Brazil. Am J Trop Med Hyg. 2008;78(1):176–8. doi: 10.4269/ajtmh.2008.78.176 18187802

[133] Shousha AT. Species-eradication: The eradication of *Anopheles gambiae* from Upper Egypt, 1942-1945. Bull World Health Organ. 1948;1(2):309. 20603927 PMC2553915

[134] White MT, Lwetoijera D, Marshall J, Caron-Lormier G, Bohan DA, Denholm I, et al. Negative cross resistance mediated by co-treated bed nets: a potential means of restoring pyrethroid-susceptibility to malaria vectors. PLoS One. 2014;9(5):e95640. doi: 10.1371/journal.pone.0095640 24788951 PMC4006834

[135] WHO. Achieving and maintaining universal coverage with long-lasting insecticidal nets for malaria control. Global Malaria Programme; 2017.

[136] Govella NJ, Ogoma SB, Paliga J, Chaki PP, Killeen G. Impregnating hessian strips with the volatile pyrethroid transfluthrin prevents outdoor exposure to vectors of malaria and lymphatic filariasis in urban Dar es Salaam, Tanzania. Parasit Vectors. 2015;8:322. doi: 10.1186/s13071-015-0937-8 26063216 PMC4465323

[137] Masalu JP, Finda M, Okumu FO, Minja EG, Mmbando AS, Sikulu-Lord MT, et al. Efficacy and user acceptability of transfluthrin-treated sisal and hessian decorations for protecting against mosquito bites in outdoor bars. Parasit Vectors. 2017;10(1):197. doi: 10.1186/s13071-017-2132-6 28427437 PMC5397833

[138] Ogoma SB, Mmando AS, Swai JK, Horstmann S, Malone D, Killeen GF. A low technology emanator treated with the volatile pyrethroid transfluthrin confers long term protection against outdoor biting vectors of lymphatic filariasis, arboviruses and malaria. PLoS Negl Trop Dis. 2017;11(4):e0005455. doi: 10.1371/journal.pntd.0005455 28388682 PMC5384659

[139] Mmbando AS, Ngowo H, Limwagu A, Kilalangongono M, Kifungo K, Okumu FO. Eave ribbons treated with the spatial repellent, transfluthrin, can effectively protect against indoor-biting and outdoor-biting malaria mosquitoes. Malar J. 2018;17(1):368. doi: 10.1186/s12936-018-2520-1 30333015 PMC6192339

[140] Swai JK, Soto AC, Ntabaliba WS, Kibondo UA, Ngonyani HA, Mseka AP, et al. Efficacy of the spatial repellent product Mosquito Shield™ against wild pyrethroid-resistant *Anopheles arabiensis* in south-eastern Tanzania. Malar J. 2023;22(1):249. doi: 10.1186/s12936-023-04674-4 37649032 PMC10466708

