# Supplementary material for: Blood host preferences and competitive inter-species dynamics within an African malaria vector species complex inferred from signs of animal activity around aquatic larval habitats
Source: PLoS One. 2026 Mar 27;21(3):e0344670. doi: 10.1371/journal.pone.0344670 (PMC13029809; doi:10.1371/journal.pone.0344670)
Supplement: S1 Appendix — The rationale for selecting camp locations which acted as the sampling frame for the study (Fig 1), and the logistics and day-to-day procedures of visiting these camps. Includes a table of camp locations, and photographs illustrating some camps as examples. (PDF) [file pone.0344670.s001.pdf]

S1 Appendix: Camp locations and field logistics.

The team of three investigators responsible for collecting data and mosquito specimens were accompanied by eight VGS, recruited from stakeholder communities that are responsible for patrolling and protecting the ILUMA WMA. The VGS in addition to providing food, water, shelter and security, were also integral to the implementation of the field protocol. Walking from one camp to the next was only possible under the guidance of at least two armed VGS members, whom also assisted with tracking for the surveys of humans, livestock and wild animals. Field procedures conducted inside NNP were also completed under the oversight and guidance of armed park rangers. Other VGS members assisted with carrying equipment and supplies, as well as the routine logistical and scientific activities such as setting up camp, cooking, filtering water for drinking, and collecting and maintaining mosquitoes before they were transported back to the central camp insectary at *Msakamba*. The field protocol was driven by the primary objective for the broader study that was to find insecticide susceptible mosquitoes (1), so all field activities, including the larval surveys, and the land use and mammalian activity surveys reported in this study were centred around the daily collection of live adult mosquitoes.

After scouting potential locations in across ILUMA WMA and considering the suggestions of the VGS based on their vast personal knowledge of the area, 28 camps were selected within and adjacent to ILUMA WMA (Table S1.1). Due to transport constraints and survey requirements for corresponding studies (2-5), almost all of the data collection process was completed on foot. Therefore, circuits were designed to manage the physical challenges of the study in the absence of vehicle support. Each circuit had a planned route that minimised walking distances between camps which ensured that the team had enough time to rest in the afternoon before proceeding with data collection in the evening and the following day.

Camps were visited consecutively for two nights each. On a *moving day*, the investigators walked from one camp to the next, and wildlife surveys were completed *en route* for a corresponding study (2). The larval surveys and associated mammal surveys conducted for this report were carried out on the following day for that camp location. Live mosquitoes were also carried out daily, in the morning and the evening for a corresponding study (3). Therefore, the circuit design for sequentially visiting camps in a rolling cross-sectional survey was crucial for sustaining optimal long-term data collection by limiting investigator fatigue and allowing them to take breaks of a few days in between periods of continuous field work that lasted about two weeks for a single circuit.

The original protocol planned for a total of three rounds to be completed from January to March, March to May, and August to October 2022, representing the short rainy season, long rainy season, and the dry season, respectively. It was planned that each round of surveys in the longitudinal rolling cross-sectional study design visited all 28 camps detailed Table S1.1, but in each round a few camps were omitted for pragmatic reasons such as a lack of surface water during the dry season or inaccessibility due to severe flooding during intense rains. Also, in the first round, the north-west circuit was omitted because transport, handling and rearing procedures for the collected mosquito specimens failed, and so it was decided to adjust these procedures and start afresh with round two.

However, no camp inside the WMA lacked any signs of human disturbance, and only a few remained relatively conserved, so four new mobile camps inside NNP were added (camps 29-32) to the study design at the end of round three in November 2022, forming a new circuit that was surveyed with vehicle support for logistical and safety reasons. These camps were located inside the boundary of NNP, immediately to the east of ILUMA WMA to capture the best

conserved environments and were accessed by vehicle via the NNP ranger post at Boma Ulanga for which that circuit was named (Table S1.1, Figure 1). These were then repeated at the start of the fourth and final round of data collection which was completed from February to July 2023, representing the whole wet season and the beginning of the dry season for that calendar year. Considering the identification of *An. quadriannulatus* and emerging results associated with a corresponding study, it was decided to extend the sampling frame deeper into the park and adjust the field protocol to collect and immediately preserve additional collections of larvae that would not be used to rear adults in the Msakamba insectary (S2 Appendix). Again, for logistical and safety reasons, additional camps located along the Kilombero river were accessed via motorboat in the rainy season and via vehicle on land during the dry season.

A list of all 40 camp locations is presented in Table S1.1. Examples of camp locations are photographed in Figures S1.2 to 1.5.

Table 1.1: Number, name, location, coordinates, and ecological characteristics of each camp location, together with the quadrant circuit to which it was assigned and the number of times it was surveyed(5, 6).

Number	Name	Circuit	Location	Coordinates	Number of times surveyed	Habitat Type
1	Msakamba	Southeast	Inside ILUMA WMA	-8.25981S, 36.85936E	4	LRecovering miombo woodland along a small seasonal stream bed.
2	Msiba wa Deo	Southeast	Inside ILUMA WMA	-8.25821S, 36.88524E	4	Intact miombo woodland along a small seasonal stream bed.
3	Bwawa la Nyati	Southeast	Inside ILUMA WMA	-8.29598S, 36.88732E	4	Intact miombo woodland surrounding a large waterhole.
4	Bwawa la Nandete	Southeast	Inside ILUMA WMA	-8.3467S, 36.9024E	4	Degraded miombo woodland surrounding a large waterhole.
5	Korongo la Bundu	Southeast	Inside ILUMA WMA	-8.37768S, 36.90163E	4	Degraded miombo woodland along a small seasonal stream bed.
6	Bwawa la Namamba	Southeast	Inside ILUMA WMA	-8.34931S, 36.81653E	4	Degraded miombo woodland surrounding a large waterhole.
7	Bwawa la Chakacheni	Southeast	Inside ILUMA WMA	-8.31649S, 36.81507E	4	Degraded miombo woodland surrounding a large waterhole.
8	Bwawa la Njuju	Northeast	Inside ILUMA WMA	-8.24204S, 36.86268E	4	Intact miombo woodland surrounding a large waterhole.
9	Bwawa la Chamvi	Northeast	Inside ILUMA WMA	-8.22176S, 36.89241E	3	Intact miombo woodland surrounding a large waterhole.
10	Bwawa la Miembeni	Northeast	Inside ILUMA WMA	-8.22401S, 36.87518E	2	Mostly intact miombo woodland surrounding a waterhole.
11	Kisima cha Seba	Northeast	Inside ILUMA WMA	-8.20749S, 36.85738E	3	Intact miombo woodland adjacent to a small waterhole.
12	Bwawa la Maya	Northeast	Inside ILUMA WMA	-8.19492S, 36.88045E	4	Transition zone between miombo woodland and groundwater forest surrounding a large waterhole.
13	Bwawa la Mrope	Northeast	Inside ILUMA WMA	-8.16321S, 36.88341E	3	Dense groundwater forest surrounding a large waterhole.
14	Mikeregembe	Northeast	Inside ILUMA WMA	-8.15048S, 36.87539E	4	Authorised fishing camp on the banks of the Kilombero river, open but bordering groundwater forest.
15	Mdalangwila	Northeast	Inside ILUMA WMA	-8.16041S, 36.83887E	4	Authorised fishing camp on the banks of the Kilombero river, open but bordering groundwater forest.

16	Bwawa la Muamachi	Southwest	Village outside western boundary of ILUMA WMA	-8.38785S, 36.74629E	4	Village outside the conservation area surrounding a large waterhole.
17	Tuliza Moyo	Southwest	Village outside western boundary of ILUMA WMA	-8.35954S, 36.74821E	4	Village outside the conservation area along a small seasonal stream bed.
18	Mavimba Porini	Southwest	Village outside western boundary of ILUMA WMA	-8.33982S, 36.74628E	4	Village outside the conservation area along a large river and surrounding a large waterhole.
19	Bwawa la Selesussi	Southwest	Inside ILUMA WMA	-8.33064S, 36.76951E	4	Degraded miombo woodland surrounding a large waterhole.
20	Makingi	Southwest	Village outside western boundary of ILUMA WMA	-8.27593S, 36.74737E	4	Village outside the conservation area along a small seasonal stream bed.
21	Bwawa la Mpunga	Southwest	Inside ILUMA WMA	-8.27426S, 36.81946E	4	Highly degraded miombo woodland surrounding a large waterhole.
22	Kisaki	Northwest	Village outside western boundary of ILUMA WMA	-8.24595S, 36.7669E	3	Largest human settlement, at the base of a large hill adjacent to a spring and large waterhole.
23	Uwanja wa Ndege	Northwest	Village outside western boundary of ILUMA WMA	-8.23448S, 36.80218E	3	Human settlement along a small seasonal river.
24	Bwawa la Mkwajuni	Northwest	Inside ILUMA WMA	-8.21681S, 36.81141E	3	Intact miombo woodland surrounding a degraded large waterhole.
25	Bwawa la Mamba Luhogi	Northwest	Inside ILUMA WMA	-8.19352S, 36.78527E	3	Intact miombo woodland surrounding a highly degraded large waterhole.
26	Funga	Northwest	Inside ILUMA WMA	-8.16924S, 36.776E	3	Authorised fishing camp on the banks of the Kilombero river, open but bordering intact groundwater forest.
27	Bwawa la Mlenda	Northwest	Inside ILUMA WMA	-8.20037S, 36.82132E	3	Intact miombo woodland surrounding a large waterhole.
28	Bwawa la Semka	Northwest	Inside ILUMA WMA	-8.20436S, 36.84023E	2	Largely intact groundwater forest

						surrounding a large waterhole.
29	Bwawa la Simba	Boma Ulanga	Nyerere NP (East of ILUMA WMA)	-8.31298S, 36.94381E	2	Transition zone of mixed miombo woodland and acacia savanna surrounding a waterhole.
30	Kiboko Zanzibar	Boma Ulanga	Nyerere NP (East of ILUMA WMA)	-8.26313S, 37.00293E	2	Open acacia savanna on the banks of the Kilombero river.
31	Zanzibar	Boma Ulanga	Nyerere NP (East of ILUMA WMA)	-8.2565S, 36.98337E	2	Transition zone of mixed miombo woodland and acacia savanna on the banks of the Kilombero river.
32	Bwawa la Moto	Boma Ulanga	Nyerere NP (East of ILUMA WMA)	-8.2741S, 36.93866E	2	Transition zone of mixed miombo woodland and acacia savanna surrounding a waterhole.
33	Kambi ya Mamba	Kilombero	Nyerere NP (East of ILUMA WMA)	-8.18872S 36.89856E	1	Acacia savanna on the banks of the Kilombero river.
34	Kambi ya Machuma	Kilombero	Nyerere NP (East of ILUMA WMA)	-8.30422S 37.11265E	1	Acacia savanna on the banks of the Kilombero river.
35	Serengeti Ndogo	Kilombero	Nyerere NP (East of ILUMA WMA)	-8.28739S 37.09637E	1	Acacia savanna on the banks of the Kilombero river.
36	Kambi ya Makutano	Kilombero	Nyerere NP (East of ILUMA WMA)	-8.40158S 37.17879E	1	Acacia savanna on the banks of the Kilombero river.
37	Kambi ya Mawe	Kilombero	Nyerere NP (East of ILUMA WMA)	-8.40511S 37.14682E	1	Acacia savanna on the banks of the Kilombero river.
38	Shughuli Kubwa	Msolwa	Nyerere NP (East of ILUMA WMA)	-8.51759S 37.3391E	1	Miombo woodland at the Kilombero, Ulanga and Luwegu river confluences
39	Bwawa la Chatu	Msolwa	Nyerere NP (East of ILUMA WMA)	-8.01597S 37.19808E	1	Transition zone of mixed miombo woodland and acacia savanna
40	Bwawa la Umeme	Msolwa	Nyerere NP (East of ILUMA WMA)	-7.9385S 37.77394E	1	Acacia savanna at the Great Ruaha, Rufiji and Ulanga river confluences.

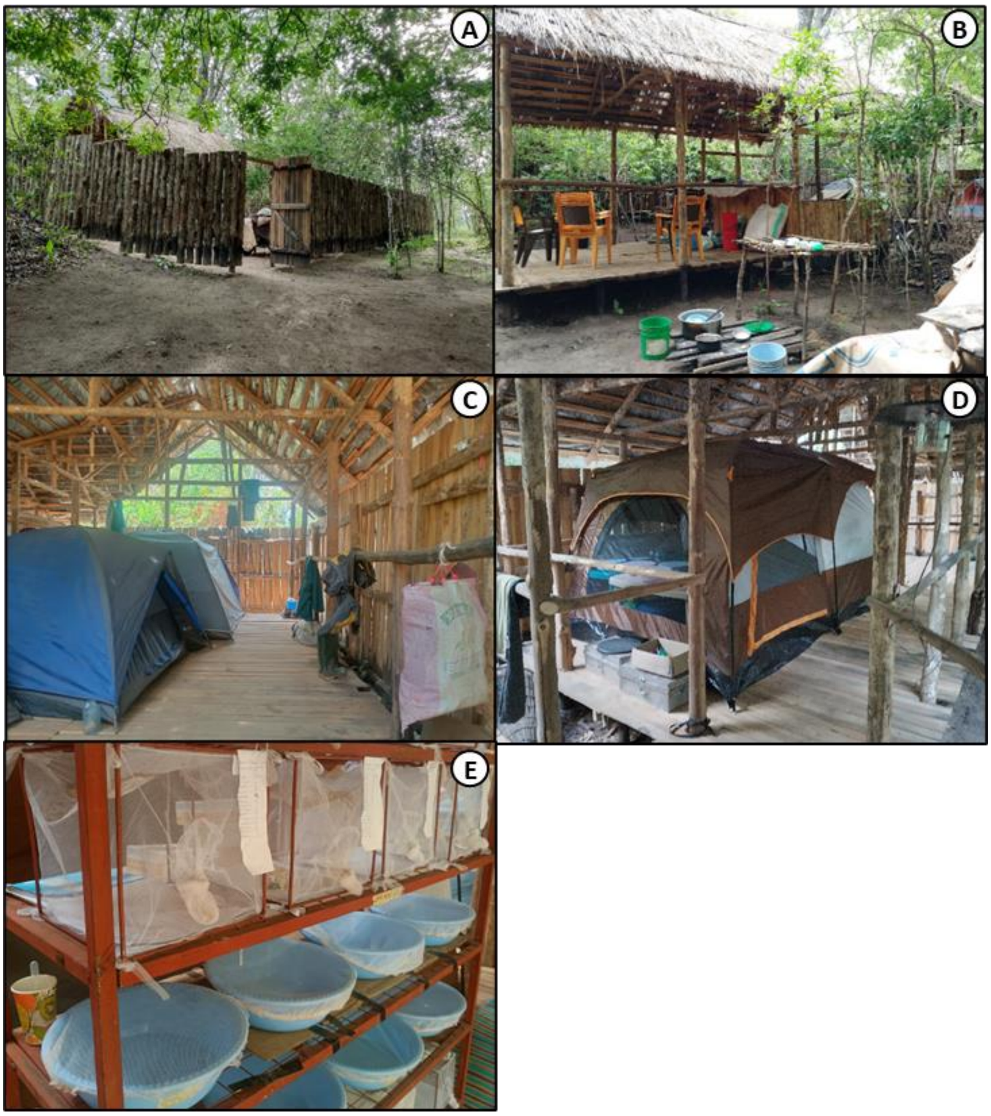

Figure S1.1: Illustrative photographs of the central camp established for the project at Msakamba. **A**: The surrounding fence for security, **B**: The kitchen and food storage area, **C**: Tents for sleeping in, **D**: A field insectary tent for housing mosquito adults and larvae, **E**; Mesh cages and plastic water basins for respectively rearing adult and larvae in the field insectary.

Msakamba is a fenced camp with essential basic infrastructure like thatch roofs, tables, chairs, large tents, a kitchen and solar-powered electricity supply was established as the hub for all scientific and logistical processes in the field.The central location of Msakamba (Figure 1) made it possible to reach any camp in ILUMA WMA within a day's walk, which ensured that live adult (for a corresponding study) and larvae samples could usually be returned in good condition within 48 hours of collection. It also ensured that food supplies, as well as recharged batteries and power packs for mobile phones and field equipment could be regularly delivered to the mobile field team moving from camp to camp every two days. Msakamba was occupied and maintained on a permanent basis by a team of Village Game Scouts (VGS) and technicians, who were responsible for maintaining the field insectary.

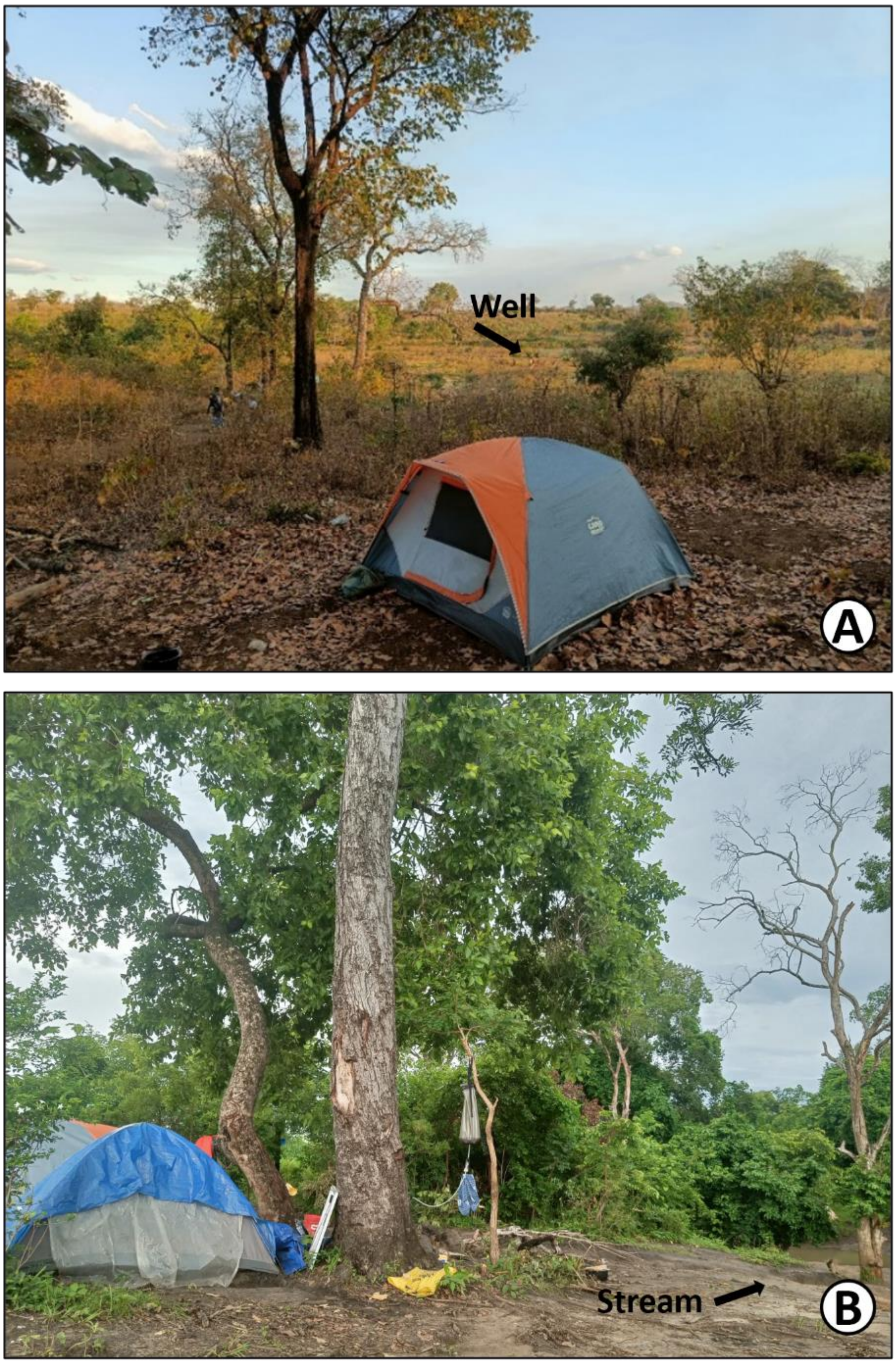

Figure S1.2: Camp number 18, *Mavimba Pori*, an example of miombo woodland that had been converted for agricultural land near the western border of ILUMA. The camp was set up close to a well during the dry season (**A**) and was located next to a flowing stream during the wet season (**B**), to fetch water for cooking and filtering drinking water. Drinking water was filtered using a MSR Autoflow XL 10L gravity water filter.

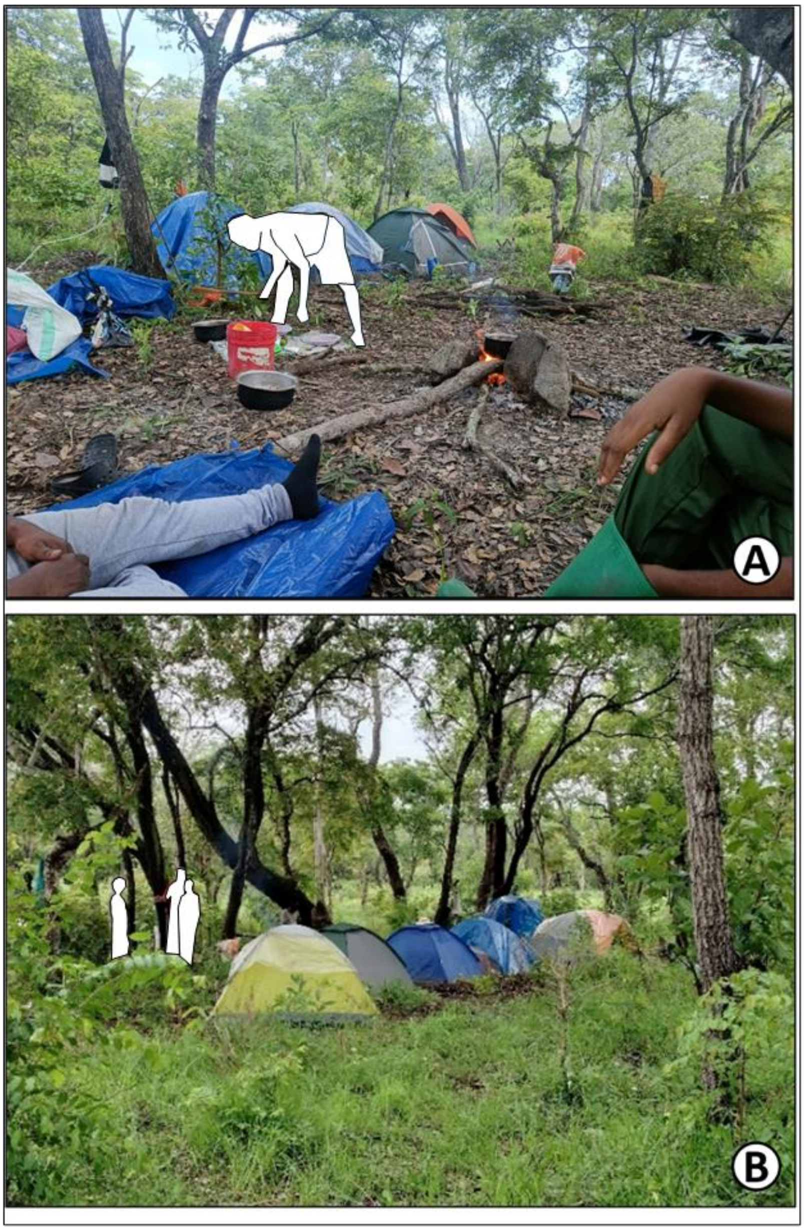

Figure S1.3: Camp number 11, *Kisima cha Seba*, located inside ILUMA WMA (A), and camp number 3, *Bwawa la Nyati* located inside ILUMA WMA and close to the NNP border. Both camps are examples of intact, mature miombo woodland.

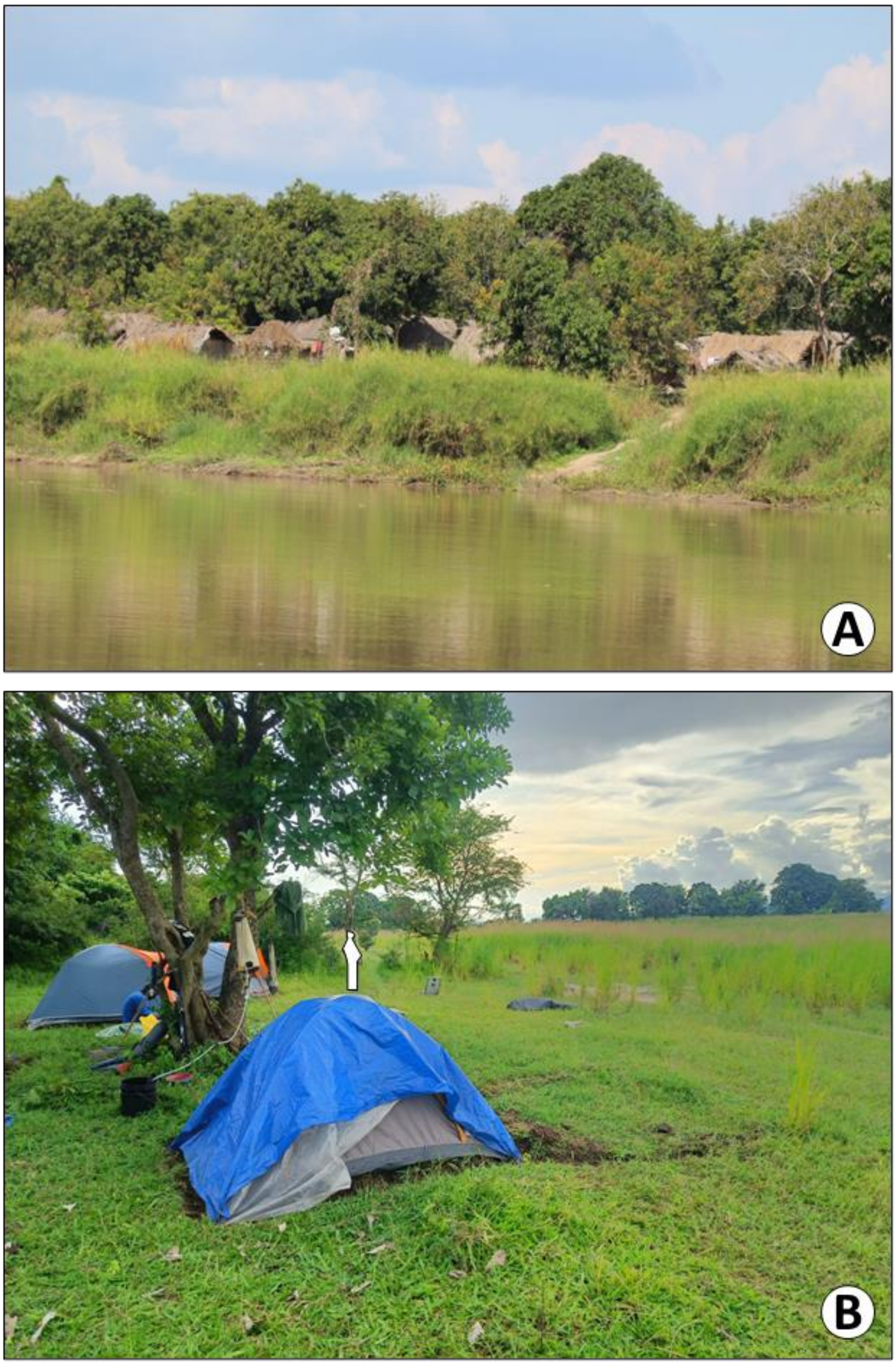

Figure S1.4: Examples of camp locations at the legal fishing camps inside the groundwater forest of ILUMA WMA on the south bank of the Kilombero, that practiced small-scale sustainable fishing. **A**; The biggest fishing camp inside ILUMA, Mikeregembe, where camp number 14 was located. **B**; Camp number 26, based next to the smaller fishing camp of Funga.

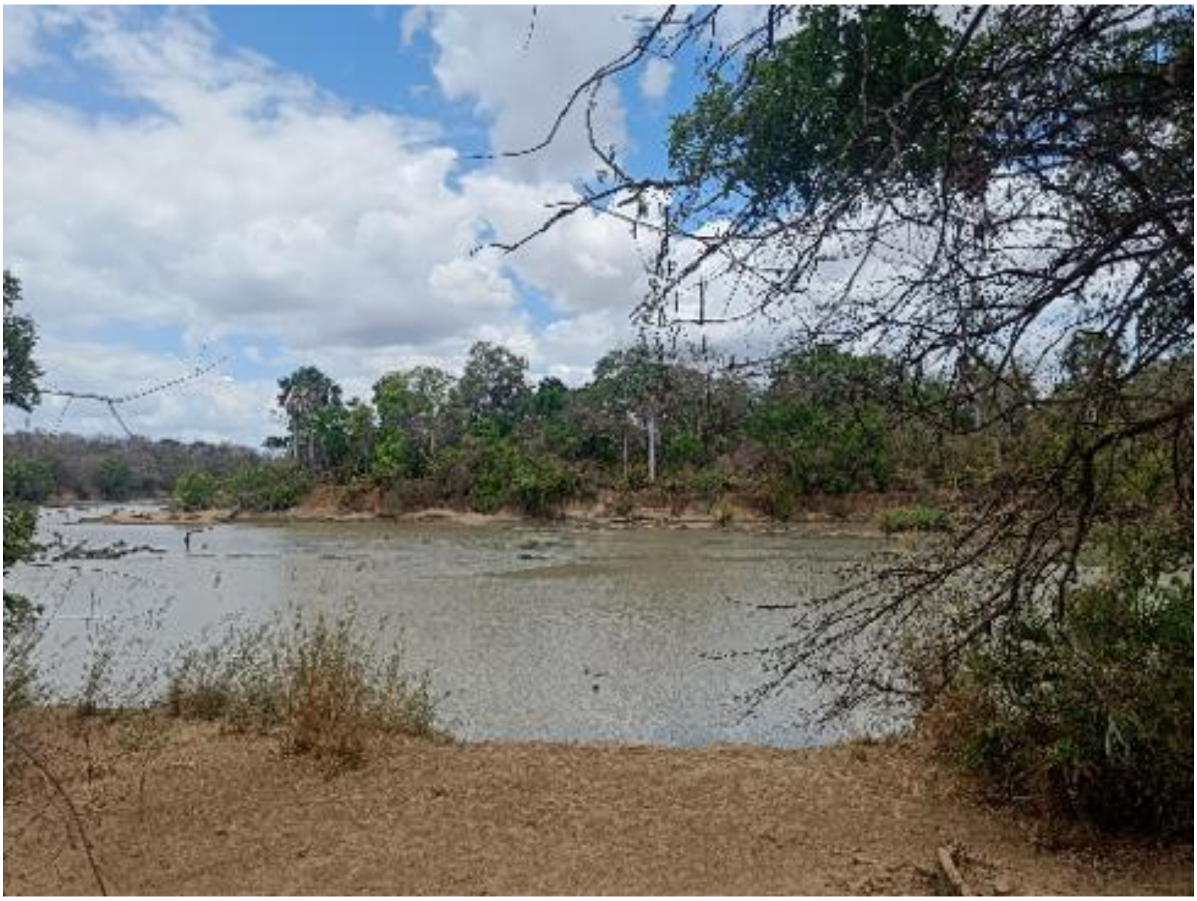

Figure S1.5: The view from camp number 31, *Zanzibar*, with a fully intact natural ecosystem inside NNP away from any signs of humans or livestock.

References

1. Killeen GF. Population-stabilizing portfolio effects of fine-scale environmental variations in natural resource availability to malaria vector mosquitoes; Characterization and implications for malaria vector control strategies: University College Cork; 2023 [Available from: https://www.ucc.ie/en/eri/projects/population-stabilizing-portfolio-effects-of-fine-scale-environmental-variations-in-natural-resource-availability-to-malaria-vector-mosquitoes-characterization-and-implications-for-malaria-vector-control-strategies.html.
2. Duggan LM, Tarimo LJ, Walsh KA, Kavishe DR, Crego R, Manase E, et al. Direct comparative assessment of radial and transect surveys to document wild mammal activity across diverse habitat types. African Journal of Ecology. 2024;62(3):e13309.
3. Kavishe D, Walsh K, Msoffe R, Duggan L, Tarimo L, Butler F, et al. Comparative attractiveness of Anopheles quadriannulatus and Anopheles arabiensis to humans estimated by comparing the relative abundance of these two species in larval samples, unbaited adult catches and human-baited adult catches. BioRxiv. 2024.
4. Kavishe DR, Msoffe RV, Malika GZ, Walsh KA, Duggan LM, Tarimo LJ, et al. A self-cooling self-humidifying mosquito carrier backpack for transporting live adult mosquitoes on foot over long distances under challenging field conditions. Medical and Veterinary Entomology. 2025;39(1):171-86.
5. Duggan LM. The influence of community-defined land use plans and de facto land use practices on the relative abundance and distribution of large wild mammals in a community-based Wildlife Management Area in Southern Tanzania. MSc (Research) Thesis: University College Cork; 2023.
6. Walsh KA. Blood host preferences and competitive inter-species dynamics within an African malaria vector species complex inferred from signs of animal activity around aquatic larval habitats distributed across a gradient of fully domesticated to fully pristine ecosystems in southern Tanzania. . MSc (Research) Thesis: University College Cork; 2023.
