# Supplementary material for: Blood host preferences and competitive inter-species dynamics within an African malaria vector species complex inferred from signs of animal activity around aquatic larval habitats
Source: PLoS One. 2026 Mar 27;21(3):e0344670. doi: 10.1371/journal.pone.0344670 (PMC13029809; doi:10.1371/journal.pone.0344670)
Supplement: S4 Appendix — (PDF) [file pone.0344670.s004.pdf]

S4 Appendix: Assessment of natural ecosystem integrity

Scoring locations in terms of a Subjective Natural Ecosystem Integrity Index (SNEII) based on consensus investigator impressions.

Following the initial year of data collection and prior to any analyses, a *subjective natural ecosystem integrity index* (SNEII) was devised exactly as described in Duggan *et al*. [1]. In summary, the index was created as a simple and intuitive means of assessing the ecological intactness of a location relative to its intact natural state and the level of degradation, if any, that had occurred there. The index was created by assigning each camp a score from 0% (fully domesticated landscape) to 100% (fully intact, natural ecosystem) based on the recollected impressions of the three investigators who had all visited the camps at least once.

Note, however, that the SNEII in this report includes an additional 8 camp locations in Nyerere National Park compared to the initial SNEII reported in *et al*. [1]. The scores were initially drafted and then finalised in Microsoft Excel® after further detailed conversations reached a conclusive consensus.

The overall outcomes of this subjective ecosystem scoring processes are detailed in table S4.1.

Table S4.1: The values and corresponding ranks for the subjective natural ecosystem integrity index (SNEII) based on a consensus of recollecSNEII ted impressions and the objective natural ecosystem integrity index (ONEII) based on principal component analysis (PCA) of natural resource use, direct sightings and signs of tracks, spoor and other signs for wildlife, livestock and humans, together with every recorded estimate of the proportion of land used for rice farming and other tillage crops.

			Subjective Ecosystem Integrity Index		Objective Ecosystem Integrity Index	
Camp	Camp Name	Location	Value [a]	Rank [b]	Value [c]	Rank [d]
1	Msakamba	Inside ILUMA	70%	16.5	-0.301	17
2	Bwawa la Msiba wa Deo	Inside ILUMA	84%	24.0	-0.361	28
3	Bwawa la Nyati	Inside ILUMA	85%	25.5	-0.323	22
4	Bwawa la Nandete	Inside ILUMA	35%	10.0	-0.283	14
5	Korongo la Bundu	Inside ILUMA	30%	9.0	-0.279	13
6	Bwawa la Namamba	Inside ILUMA	40%	11.0	-0.266	12
7	Bwawa la Chakacheni	Inside ILUMA	15%	7.0	-0.255	10
8	Bwawa la Njuju	Inside ILUMA	81%	21.0	-0.325	24
9	Bwawa la Chamvi	Inside ILUMA	86%	27.0	-0.353	26
10	Bwawa la Miembeni	Inside ILUMA	68%	15.0	-0.397	30
11	Kisima cha Seba	Inside ILUMA	82%	22.0	-0.293	16
12	Bwawa la Maya	Inside ILUMA	87%	28.0	-0.359	27
13	Bwawa la Mrope	Inside ILUMA	90%	29.0	-0.325	23
14	Mikeregembe	Inside ILUMA	75%	19.0	-0.311	20
15	Mdalangwila	Inside ILUMA	72%	18.0	-0.310	19
16	Bwawa la Mnyuamachi	Outside ILUMA	6%	5.0	1.185	5
17	Tuliza Moyo	Outside ILUMA	4%	3.0	1.219	4
18	Mavimba Pori	Outside ILUMA	10%	6.0	0.158	7
19	Bwawa la Selesusi	Inside ILUMA	25%	8.0	-0.253	9
20	Makingi	Outside ILUMA	0%	1.0	1.286	3
21	Bwawa la Mpunga	Inside ILUMA	55%	14.0	-0.263	11

22	Kisaki	Outside ILUMA	5%	4.0	4.722	1
23	Uwanja wa Ndege	Outside ILUMA	3%	2.0	2.750	2
24	Bwawa la Mkwajuni	Inside ILUMA	45%	12.0	-0.138	8
25	Bwawa la Mamba Luhogi	Inside ILUMA	48%	13.0	0.391	6
26	Funga	Inside ILUMA	77%	20.0	-0.287	15
27	Bwawa la Mlenda	Inside ILUMA	83%	23.0	-0.306	18
28	Bwawa la Semka	Inside ILUMA	85%	25.5	-0.382	29
29	Kambi ya Simba	Inside NNP	97%	32.0	-0.478	36
30	Bwawa la Kiboko Zanzibar	Inside NNP	100%	37.5	-0.520	40
31	Zanzibar	Inside NNP	95%	30.5	-0.458	35
32	Bwawa la Moto	Inside NNP	100%	37.5	-0.513	39
33	Kambi ya Mamba	Inside NNP	98%	33.0	-0.415	32
34	Kambi ya Machuma	Inside NNP	100%	37.5	-0.500	37
35	Serengeti Ndogo	Inside NNP	95%	30.5	-0.451	34
36	Kambi ya Makutano	Inside NNP	100%	37.5	-0.446	33
37	Kambi ya Mawe	Inside NNP	100%	37.5	-0.504	38
38	Shughuli kubwa	Inside NNP	100%	37.5	-0.339	25
39	Bwawa la Chatu	Inside NNP	99%	34.0	-0.399	31
40	Bwawa la Umeme	Inside NNP	70%	16.50	-0.315	21

[a] The subjective natural ecosystem integrity score.
[b] The rank of each camp based on the subjective ecosystem integrity scores, where the lowest rank represents fully degraded, domesticated land and the highest rank represents an absolute intact natural ecosystem.
[c] PC1 values that were derived from a PCA accounting for all recorded detections of humans, livestock and wildlife, and land use activities that were standardised using z-scores.
[d] The rank of each camp based on standardised PC1 values, where the lowest rank represents the most degraded camp and the highest rank represents the best conserved camp.

For interpretation of the SNEII values for camps 1 to 32, see Duggan et al. (2025) [1]. For the additional camps in this report, 7/8 camps have scores of >95%. The lowest score inside NNP was assigned to one camp located 42km inside the park boundary. A score of 70% was given to camp number 40, *Bwawa la Umeme* because the area around this camp was recently cleared in relation to the Julius Nyerere Hydropower Station located on the Rufiji River (Figure 1). Despite this evidence of human intervention, large proportions of the area around the camp were in good condition and personal observations indicated that wildlife was abundant and diverse, albeit less so than in any other NNP camp.

Validation of the SNEII by comparison an Objective Natural Ecosystem Integrity Index (ONEII) based on statistical synthesis of formal quantitative surveys of human, livestock and wildlife activities, as well as land cover attributes

To assess the validity of this intuitive approach to quantifying ecosystem integrity, this subjective index was tested for correlation with an alternative, *objective natural ecosystem integrity index* (ONEII), based on a principal component analysis (PCA) of values for all the various types of detections of human, livestock and wildlife activity and land use [1].

The methodology for obtaining the ONEII scores for camps 1-40 was followed exactly as described in Duggan et. al. (2025) [1], and the results are presented in Table S4.1.

A Spearman's rank correlation test was then used to compare the ONEII with the SNEII [1], and the results are presented in Figure S4.1

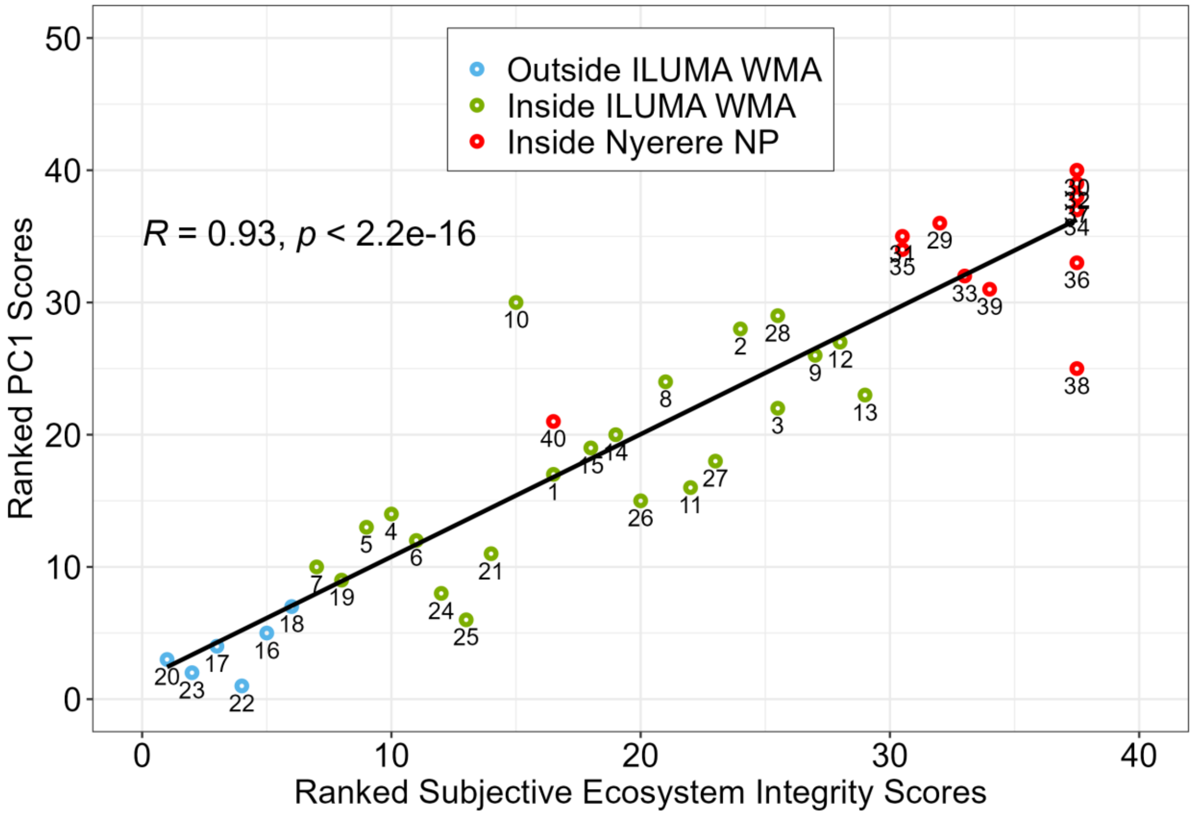

Figure S4.1. The ranked standardised PC1 values of the ONEII for each camp number plotted against the ranked SNEII scores for each camp. Note that while this graph is almost identical to that in Duggan *et al.* [1], it includes 8 extra camps, one of which (number 40), is a useful exception with unusually low ecosystem integrity for a location so far inside NNP which helps to prove the general rule of close correlation.

Apart from two moderate outliers, *Shuguli kubwa* (camp number 36) and *Bwawa la Miembeni* (camp number 10), that were respectively ranked lower and higher compared to SNEII ranks, the high correlation displayed in Figure S4.1 indicates that this novel methodology of formulating an ecosystem integrity index based on repeated recollective impressions of the study sites was a highly effective approach, and even has advantages compared to the use of the ONEII. The more convenient and intuitive SNEII is measured on a scale from 0% to 100%, thus providing a simplified interpretation of the ecological state at each study site where larvae surveys were conducted. Furthermore, multivariate regression analysis demonstrated that the ONEII was far less sensitive than the SNEII to the observed occurrence and intensity of various

human activities [1], Throughout this study, the SNEII was therefore consistently used as the sole synthetic indicator of natural ecosystem integrity in the analyses of larval occupancy (S6 Appendix) and the association between environmental parameters and *An. gambiae* complex sibling species composition (S6 Appendix).

References

1. Duggan L, Walsh K, Tarimo L, Kavishe D, Crego R, Manase E, et al. A subjective and intuitive approach to rapid, holistic assessment of natural ecosystem integrity across a community-managed conservation area in southern Tanzania. Ecology and Evolution. 2025;15(3):e70872.
2. Service M. Mosquito (Diptera: Culicidae) dispersal—the long and short of it. Journal of medical entomology. 1997;34(6):579-88.
