# Supplementary material for: Blood host preferences and competitive inter-species dynamics within an African malaria vector species complex inferred from signs of animal activity around aquatic larval habitats
Source: PLoS One. 2026 Mar 27;21(3):e0344670. doi: 10.1371/journal.pone.0344670 (PMC13029809; doi:10.1371/journal.pone.0344670)
Supplement: S9 Fig — (PDF) [file pone.0344670.s009.pdf]

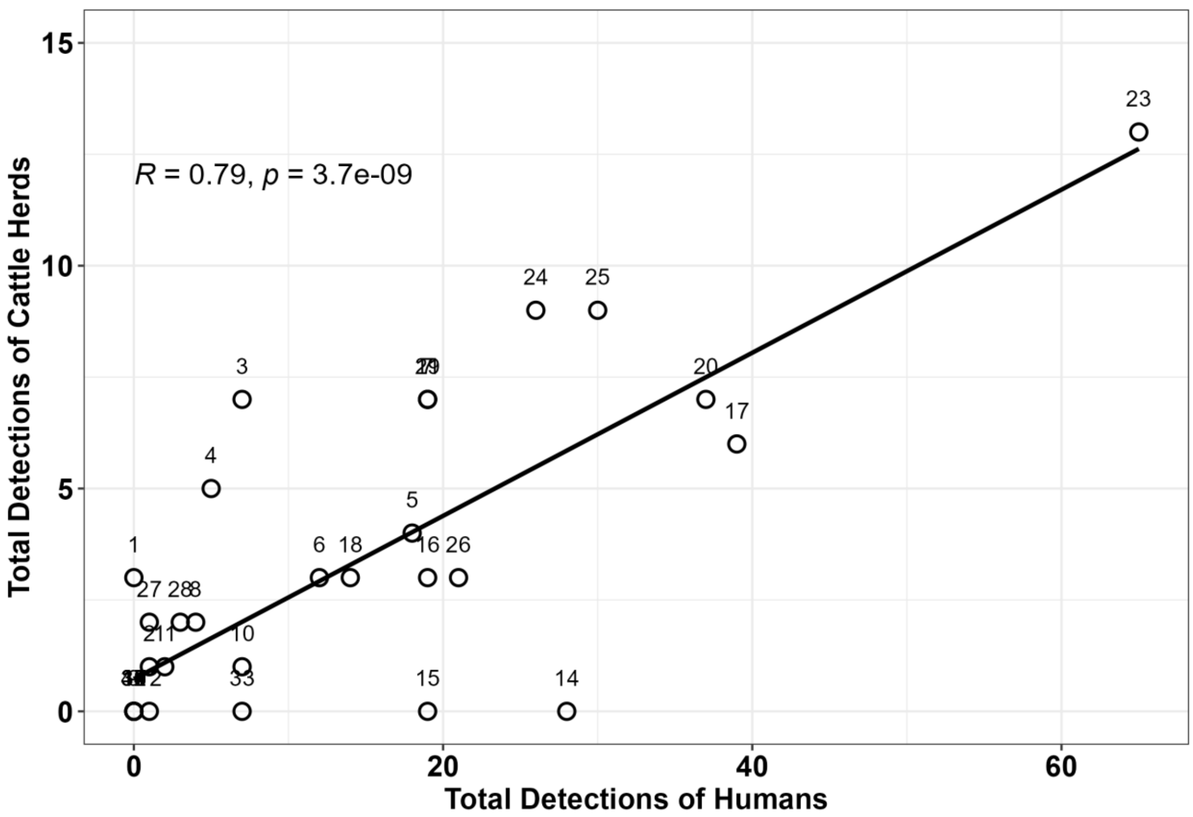

S9 Figure. The detection frequency of humans plotted against the detection frequency of cattle herds at each camp number demonstrating a strong linear correlation, as tested using a Pearson's linear correlation test.
