# Supplementary material for: Blood host preferences and competitive inter-species dynamics within an African malaria vector species complex inferred from signs of animal activity around aquatic larval habitats
Source: PLoS One. 2026 Mar 27;21(3):e0344670. doi: 10.1371/journal.pone.0344670 (PMC13029809; doi:10.1371/journal.pone.0344670)
Supplement: S11 Text — (PDF) [file pone.0344670.s011.pdf]

S11 Text. Defining competition, competitive displacement and competitive co-existence in strict ecological terms.

Competition occurs when two species require the same limiting resources [1, 2]. The superior competitor reduces the availability of resources, mainly through exploitative or interference mechanisms [3, 4], thus weakening the survival and reproductive success of the latter competitor. Therefore, in a competitive relationship between two species, the relative abundance of one species is affected by the abundance of its competitor [5, 6], just as illustrated for *An. arabiensis* and *An. quadriannulatus* in Figure 3. The most extreme outcome of competition is known as *competitive displacement* [7], when one species exhibits such a clear competitive advantage that its dominance leads to the elimination of the other. Hence, this concept is based on the principle of competitive exclusion [8], wherein it is assumed, sometimes too simplistically, that two species can only co-exist if they compete for different limiting resources [8, 9].

However, complete competitive displacement is rarely observed between naturally co-occurring species that seemingly require the same resources. A famous example is that of the 'paradox of the plankton', where Hutchinson [10] raises the question as to why there is such diversity of co-existing plankton, despite apparently requiring the same limiting resources. Indeed, although such *competitive co-existence* sounds counter-intuitive, it can be explained by stochastic dynamics driven by external environmental fluctuations [10, 11], as well as intrinsic chaos [12, 13] and meso-scale spatial heterogeneity [14], so that ecosystem equilibrium is never reached and no single species can dominate [10].

Similarly, *Anopheles* populations are often found in sympatry [15], with aquatic stages sharing the same habitats [15-18], where larval stages need to compete for limiting resources such as nutrients and space [19-23].While *Anopheles* larval habitats certainly exhibit the kind of stochastic dynamics considered to enable competitive co-existence of two or more species [10, 24, 25], it is also worth noting that the competitive balance between species within larval populations is clearly influenced by the local abundance [16, 26] and safe accessibility [27-31] of suitable blood hosts for the relevant adult populations to feed upon. The influence of such factors, particularly the safe accessibility of blood meals, on the competitive dynamics and population composition of co-existing mosquito species are considered further in the discussion and are emphasized by Lounibos (2007). Indeed, Lounibos (2007) concludes that the dominant competitor reduces the population abundance of the weaker competitor mainly through larval resource or interference competition mechanisms, but also that complete displacement [7] between competing mosquito species does not tend to occur. Empirical field observations of sympatric populations of more than one mosquito species in most locales, even though they clearly share habitats and resources therein, therefore, also deviates from the classical but simplistic principle of competitive exclusion [8, 9], and instead resembles competitive co-existence like that observed between plankton species [10].

References

1. Tilman D. Resource competition and community structure: Princeton university press; 1982.
2. Grover JP. Resource competition: Springer Science & Business Media; 1997.
3. Schoener TW. Field experiments on interspecific competition. The american naturalist. 1983;122(2):240-85.
4. Aschehoug ET, Brooker R, Atwater DZ, Maron JL, Callaway RM. The mechanisms and consequences of interspecific competition among plants. Annual Review of Ecology, Evolution, and Systematics. 2016;47:263-81.
5. Volterra V. Variations and fluctuations of the number of individuals in animal species living together. Animal ecology. 1931:412-33.
6. Lotka AJ. The growth of mixed populations: two species competing for a common

food supply:. Journal of the Washington Academy of Sciences. 1932;22:461–9.
7. DeBach P. The competitive displacement and coexistence principles. Annual review of entomology. 1966;11(1):183-212.
8. Hardin G. The competitive exclusion principle: an idea that took a century to be born has implications in ecology, economics, and genetics. science. 1960;131(3409):1292-7.
9. Gause GF. The struggle for Existence. Baltimore: Williams and Wilkins; 1934.
10. Hutchinson GE. The paradox of the plankton. The American Naturalist. 1961;95(882):137-45.
11. Richerson P, Armstrong R, Goldman CR. Contemporaneous disequilibrium, a new hypothesis to explain the „Paradox of the Plankton". Proceedings of the National Academy of Sciences. 1970;67(4):1710-4.
12. Scheffer M, Rinaldi S, Huisman J, Weissing FJ. Why plankton communities have no equilibrium: solutions to the paradox. Hydrobiologia. 2003;491:9-18.
13. Roy S, Chattopadhyay J. Towards a resolution of 'the paradox of the plankton': A brief overview of the proposed mechanisms. Ecological complexity. 2007;4(1-2):26-33.
14. Bracco A, LaCasce J, Provenzale A. Velocity probability density functions for oceanic floats. Journal of physical oceanography. 2000;30(3):461-74.
15. Gillies MT, Coetzee M. A supplement to the Anophelinae of Africa South of the Sahara. Publ S Afr Inst Med Res. 1987;55:1-143.
16. Charlwood J, Edoh D. Polymerase chain reaction used to describe larval habitat use by Anopheles gambiae complex (Diptera: Culicidae) in the environs of Ifakara, Tanzania. Journal of Medical Entomology. 1996;33(2):202-4.
17. Minakawa N, Mutero CM, Githure JI, Beier JC, Yan G. Spatial distribution and habitat characterization of anopheline mosquito larvae in Western Kenya. The American journal of tropical medicine and hygiene. 1999;61(6):1010-6.
18. Gimnig JE, Ombok M, Kamau L, Hawley WA. Characteristics of larval anopheline (Diptera: Culicidae) habitats in Western Kenya. Journal of medical entomology. 2001;38(2):282-8.
19. Koenraadt C, Majambere S, Hemerik L, Takken W. The effects of food and space on the occurrence of cannibalism and predation among larvae of Anopheles gambiae sl. Entomologia Experimentalis et Applicata. 2004;112(2):125-34.
20. Service M. Mosquito ecology: field sampling methods. Second ed. Hall C, editor: Springer; 1993.
21. Dye C. Competition amongst larval Aedes aegypti: the role of interference. 1984.
22. Barbosa P, Peters TM, Greenough N. Overcrowding of mosquito populations: responses of larval Aedes aegypti to stress. Environmental Entomology. 1972;1(1):89-93.
23. Carpenter SR. Resource limitation of larval treehole mosquitoes subsisting on beech detritus. Ecology. 1983;64(2):219-23.
24. Chesson PL. Environmental variation and the coexistence of species. Community ecology. 1986;240:54.
25. Chesson P. General theory of competitive coexistence in spatially-varying environments. Theoretical population biology. 2000;58(3):211-37.

26. Minakawa N, Seda P, Yan G. Influence of host and larval habitat distribution on the abundance of African malaria vectors in western Kenya. The American journal of tropical medicine and hygiene. 2002;67(1):32-8.
27. Gillies MT, Smith A. The effect of a residual house-spraying campaign in East Africa on species balance in the Anopheles funestus group. The replacement of A. funestus Giles by A. rivulorum Leeson. Bulletin of Entomological Research. 1960;51(2):243-52.
28. Smith A. Malaria in the Taveta Area of Kenya and Tanganyika. Part III. Entomological Findings Three Years after the Spraying Period. East African medical journal. 1962;39(9):553-64.
29. Bayoh MN, Mathias DK, Odiere MR, Mutuku FM, Kamau L, Gimnig JE, et al. Anopheles gambiae: historical population decline associated with regional distribution of insecticide-treated bed nets in western Nyanza Province, Kenya. Malaria journal. 2010;9(1):1-12.
30. Mutuku FM, King CH, Mungai P, Mbogo C, Mwangangi J, Muchiri EM, et al. Impact of insecticide-treated bed nets on malaria transmission indices on the south coast of Kenya. Malaria journal. 2011;10(1):1-14.
31. Kawada H, Dida GO, Sonye G, Njenga SM, Mwandawiro C, Minakawa N. Reconsideration of Anopheles rivulorum as a vector of Plasmodium falciparum in western Kenya: some evidence from biting time, blood preference, sporozoite positive rate, and pyrethroid resistance. Parasites & vectors. 2012;5:1-8.
