# Supplementary material for: Blood host preferences and competitive inter-species dynamics within an African malaria vector species complex inferred from signs of animal activity around aquatic larval habitats
Source: PLoS One. 2026 Mar 27;21(3):e0344670. doi: 10.1371/journal.pone.0344670 (PMC13029809; doi:10.1371/journal.pone.0344670)
Supplement: S12 Fig — (PDF) [file pone.0344670.s012.pdf]

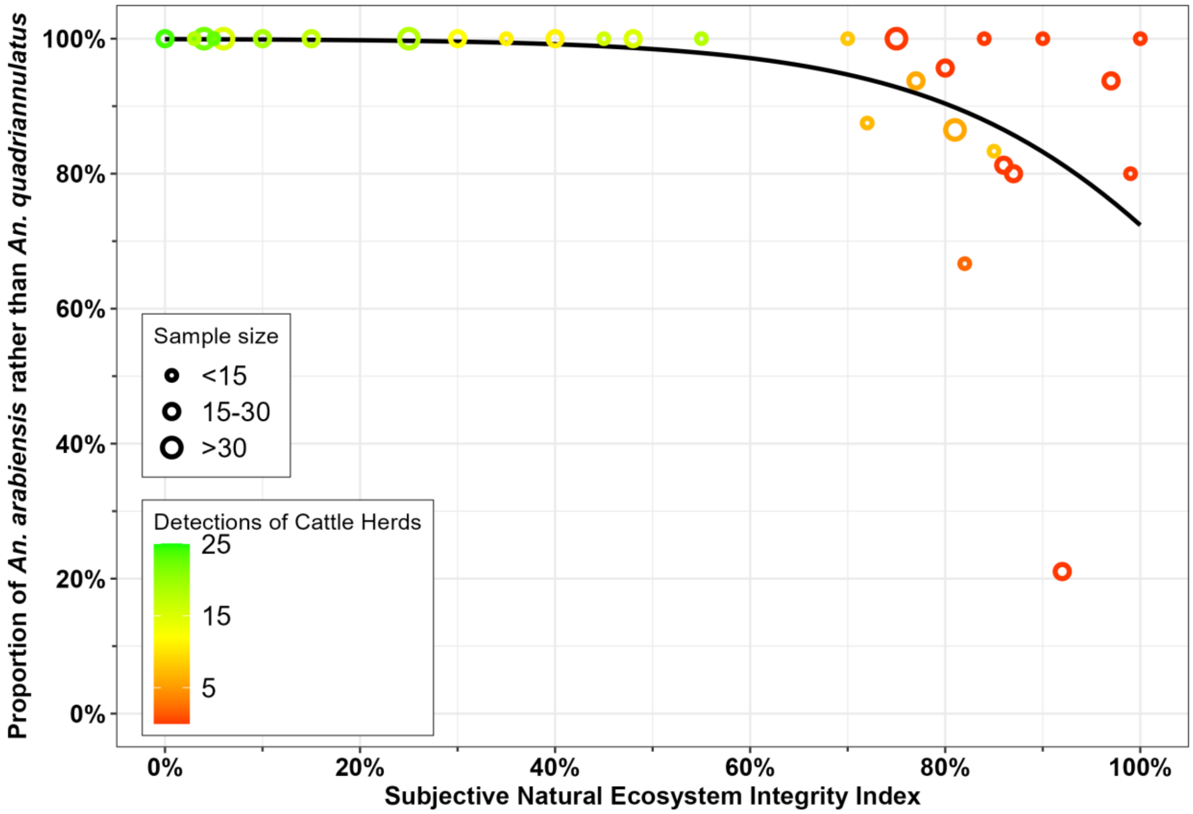

Figure S2: A scatterplot graph of the proportion of *An. gambiae* complex F_0 adults raised from larvae collected over survey rounds 1 to 3 that were identified as *An. arabiensis* rather than *An. quadriannulatus* by PCR amplification (1). Crude proportions of *An. arabiensis* for each camp are plotted against the Subjective Natural Ecosystem Integrity Index (SNEII; [(2, 3), S6 Appendix]). Sample size and the frequency with which cattle herds were detected are represented by symbol size and colour, respectively. The graph was generated with *ggplot* in R and the trendline was fitted with the *glm* option using the *geom_smooth* command, specifying a binomial distribution and logit link function for the dependent variable.

References

1. Scott JA, Brogdon WG, Collins FH. Identification of single specimens of the Anopheles gambiae complex by the polymerase chain reaction. The American journal of tropical medicine and hygiene. 1993;49(4):520-9.
2. Duggan LM. The influence of community-defined land use plans and de facto land use practices on the relative abundance and distribution of large wild mammals in a community-based Wildlife Management Area in Southern Tanzania. MSc (Research) Thesis: University College Cork; 2023. Available from: https://hdl.handle.net/10468/15928
3. Walsh KA. Blood host preferences and competitive inter-species dynamics within an African malaria vector species complex inferred from signs of animal activity around aquatic larval habitats distributed across a gradient of fully domesticated to fully pristine ecosystems in southern Tanzania. . MSc (Research) Thesis: University College Cork; 2023. Available from: https://hdl.handle.net/10468/15926
